# Functional characterization of the circadian clock in the Antarctic krill, *Euphausia superba*

**DOI:** 10.1038/s41598-017-18009-2

**Published:** 2017-12-18

**Authors:** Alberto Biscontin, Thomas Wallach, Gabriele Sales, Astrid Grudziecki, Leonard Janke, Elena Sartori, Cristiano Bertolucci, Gabriella Mazzotta, Cristiano De Pittà, Bettina Meyer, Achim Kramer, Rodolfo Costa

**Affiliations:** 1 Charité-Universitätsmedizin Berlin, Laboratory of Chronobiology, D-10117 Berlin, Germany; 2 Department of Biology, University of Padova, 35121 Padova, Italy; 3 Department of Life Sciences and Biotechnology, University of Ferrara, 44121 Ferrara, Italy; 4 Alfred Wegener Polar Biological Oceanography, 27570 Bremerhaven, Germany; 5 Carl von Ossietzky University of Oldenburg, Institute for Chemistry and Biology of the Marine Environment, 26129 Oldenburg, Germany; 6 Helmholtz Institute for Functional Marine Biodiversity Oldenburg (HIFMB), 26129 Oldenburg, Germany

## Abstract

Antarctic krill (*Euphausia superba*) is a key species in Southern Ocean ecosystem where it plays a central role in the Antarctic food web. Available information supports the existence of an endogenous timing system in krill enabling it to synchronize metabolism and behavior with an environment characterized by extreme seasonal changes in terms of day length, food availability, and surface ice extent. A screening of our transcriptome database “KrillDB” allowed us to identify the putative orthologues of 20 circadian clock components. Mapping of conserved domains and phylogenetic analyses strongly supported annotations of the identified sequences. Luciferase assays and co-immunoprecipitation experiments allowed us to define the role of the main clock components. Our findings provide an overall picture of the molecular mechanisms underlying the functioning of the endogenous circadian clock in the Antarctic krill and shed light on their evolution throughout crustaceans speciation. Interestingly, the core clock machinery shows both mammalian and insect features that presumably contribute to an evolutionary strategy to cope with polar environment’s challenges. Moreover, despite the extreme variability characterizing the Antarctic seasonal day length, the conserved light mediated degradation of the photoreceptor *Es*CRY1 suggests a persisting pivotal role of light as a *Zeitgeber*.

## Introduction

Antarctic krill (*Euphausia superba*), further named krill, is a key species in Southern Ocean food webs^1^, and plays a central role in ecosystem processes and community dynamics of apex predators^2^. While krill constitute one of the largest known underexploited stocks, with an estimated biomass of ca. 300 million tons^3^, it is still the biggest fishery by tonnage in the Southern Ocean^4^. The Southern Ocean ecosystem appears to be particularly susceptible to the recent climate changes that led to altered sea ice dynamics and a decrease in phytoplankton productivity^5,6^. Due to the central position of krill in this “wasp-waist” ecosystem^7^, their decline would chronically impact biological fluxes of energy and nutrients in the Southern Ocean. Krill exhibit rhythmic behavioral and physiological features on a daily and seasonal scale in the Southern Ocean. On the daily scale krill perform, as many other zooplankton organisms, a diel vertical migration (DVM) in the water column^8,9^. A characterization of the diurnal gene expression pattern of krill during the Antarctic summer revealed that important processes most likely related to daily environmental changes (such as energy and metabolic process, visual transduction, and stress response) exhibited certain rhythmicity. These observations underline that a circadian regulation of krill metabolism and physiology occurs under natural conditions^10^. Moreover, significant oscillations of several novel opsins with different spectral sensitivity were identified, suggesting that an endogenous mechanism modulates the complex behavioral responses of krill to changes in illumination during the daily vertical migration and during the seasonal changes of sun irradiance^11^.

Several studies^12–14^ demonstrated a food independent oscillation in feeding behaviour and metabolic activity throughout the year suggesting that other environmental factors such as photoperiod (day length) might regulate krill metabolism and behaviour also at seasonal scale. Long-term laboratory experiments demonstrate that specific krill physiological functions such as feeding^15^, metabolic activity, oxygen uptake^16^, and development^17,18^ show seasonal rhythms, controlled by an endogenous timing system (endogenous clock) in which photoperiod acts as a main *Zeitgeber* (entrainment cue), synchronizing the clock with the external world.

A prerequisite to unravel the role of an endogenous timing system in synchronising daily and seasonal behavioural patterns and physiological functions is the characterisation of the clock machinery itself. Endogenous clocks are based on overlapping molecular feedback loops that generate a self-sustained molecular oscillation of about 24 hours continuously synchronized by environmental signals. Despite the overall conservation of the molecular clock’s main components in animals (*clock*, *cycle/bmal*, *period*, and *cryptochrome*), their functions and interactions have been modelled during taxa evolution giving rise to different clock’s molecular architectures^19^. However, our current understanding of biological clocks is largely restricted to solar-controlled circadian and seasonal rhythms in terrestrial model species such as the fruit fly, the mouse or the thale cress^20^. Among Crustacea, the Atlantic horseshoe crab *Limulus polyphemus* ^21^, the copepods *Calanus finmarchicus* ^22^ and *Tigriopus californicus* ^23^ possess putative orthologues of core and accessory clock genes. The high level of conservation with known circadian clock genes suggests their involvement in the biological clock systems of these species. In other species such as the beach amphipod *Talitrus saltator* ^24,25^, the burrowing decapod *Nephrops norvegicus* ^26^, the giant river prawn *Macrobrachium rosenbergii* ^27^ and the oriental river prawn *Macrobrachium nipponense* ^28^, the rhythmic expression of at least some of the identified putative clock genes has further reinforced the hypothesis about the existence of a molecular circadian regulatory mechanism in these species. A more in-depth analysis of the circadian system has been performed in species such as the cladoceran *Daphnia pulex* ^29^, the crayfish *Procambarus clarkii* ^30^ and the intertidal isopod *Eurydice pulchra* ^31^. In particular, functional assays performed in S2 *Drosophila* cells, confirmed a transcriptional role for *Ep*CLK and *Ep*BMAL1 and an inhibitory one for *Ep*CRY2 in the *Eurydice*’s circadian mechanism^31^.

A first step toward the definition of a molecular clock model in krill was taken by Mazzotta *et al*.^32^ who identified a *cryptochrome* gene (here after termed *Es*CRY2), one of the cardinal components of the clock-work machinery in several terrestrial organisms.

Our recently published most advanced genetic database on *E. superba* (KrillDB^33^, http://krilldb.bio.unipd.it), allowed us to identify and clone sequences of the orthologues of the main circadian clock components, including *clock, cycle, period, timeless*, and *cryptochrome 1*. Using comprehensive *in silico* analyses, extensive functional characterization and study of the transcriptional temporal profile of the main clock components we unveiled the mechanism underlying the circadian endogenous clock in this key marine species and broaded our understanding of the evolutionary dynamics which have modelled the molecular oscillators during crustaceans speciation.

## Results

**Figure 1:**
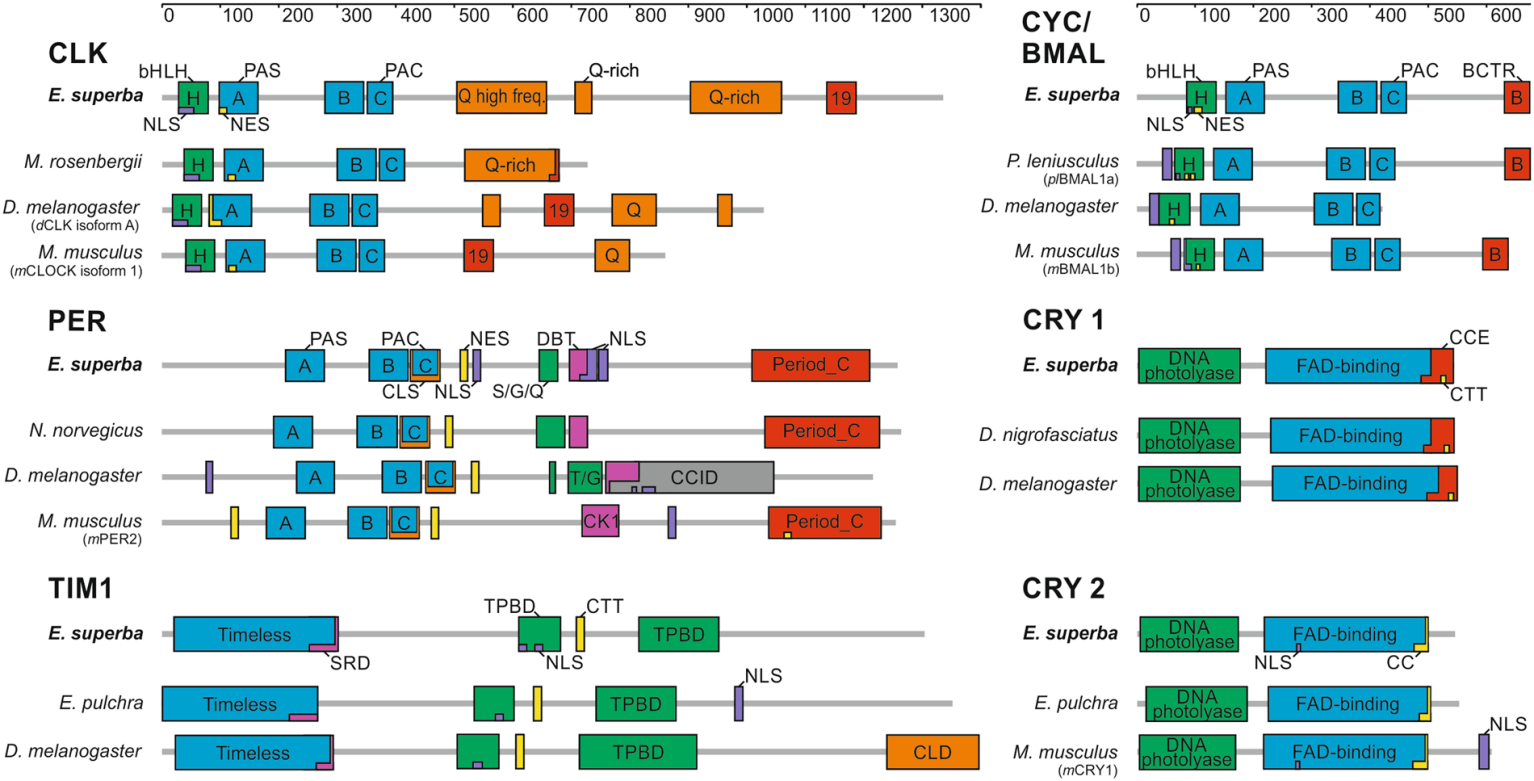
Schematic presentation of functional domains and motifs of the main krill circadian clock components (CLOCK, CYC/BMAL, PERIOD, TIMELESS 1; CRYPTOCRHOME 1, and CRYPTOCRHOME 2). Domains structure of *E. superba* proteins was compared to *D. melanogaster*, *M. musculus*, and the most similar orthologue from Crustacea. Grey bars indicate amino acidic length sequence. Specific domains were demarcated according to the SMART protein domain analysis. *Es*CLK’s exon 19 sequence corresponds to the entire exon 19 sequence of *m*CLOCK isoform 1. *Es*CYC/BMAL’s BCTR domain was defined as the final 39 amino acids of *m*BMAL1. *Es*PER’s Doubletime/Casein kinase 1 binding domain (DBT/CK1), *Es*TIM1’s serine-rich domain, and the TIM1/PER binding domains were defined via alignment to *D. melanogaster* orthologues. *Es*TIM1’s CLD corresponds to the sequence identified by deletion mutant mapping of *d*TIM^45^. *Es*CRY1 C-terminal Extension (CCE) and *Es*CRY2 Coiled-coil domain (CC) were defined by alignment to the corresponding sequence of *d*CRY1 and *m*CRY1, respectively.

Screening of the online krill transcriptome database^33^ identified orthologues of the main circadian clock components, including *clock, cycle, period, timeless*, and *cryptochrome 1* (Fig. 1, Table 1, and Supplementary Table 1 for the complete list).

**Table 1:** Domain-by-domain comparison between *E. superba* main circadian clock components and the most relevant orthologues. Peptide sequences for EsCLK, EsCYC/BMAL, EsPER, EsTIM1, EsCRY1 and EsCRY2 were aligned versus their orthologues from D. melanogaster, M. musculus, and the most related crustaceans using the EMBOSS’s online tools. For each comparison, identity/similarity percentages are reported. EsCLK’s exon 19 sequence corresponds to the entire exon 19 sequence of mCLOCK isoform 1. EsCYC/BMAL’s BCTR domain was defined as the final 39 amino acids of mBMAL1. EsPER’s Doubletime/Casein kinase 1 binding domain (DBT/CK1), EsTIM1’s serine-rich domain, and the TIM1/PER binding domains were defined via alignment to D. melanogaster orthologues. EsTIM1’s CLD corresponds to the sequence identified by deletion mutant mapping of dTIM^45^. EsCRY1 C-terminal Extension (CCE) and EsCRY2 Coiled-coil domain (CC) were defined by alignment to the corresponding sequence of dCRY1 and mCRY1, respectively.

*Es*CLOCK	*M. rosembergii*	*D. melanogaster* (*d*CLK iso.A)	*M. musculus* (*m*CLOCK iso.1)	*Es*TIMELESS 1	*E. pulchra*	*D. melanogaster*
Full-length protein	36/43	27/37	26/36	Full-length protein	42/57	25/41
HLH (28–78 aa)	90/96	67/86	61/74	Timeless (22–295 aa)	55/69	33/54
PASA (97–163 aa)	85/92	48/60	43/64	SDR (253–298 aa)	66/78	67/91*
PASB (277–343 aa)	90/100	78/87	76/85	TPBD1 (607–680 aa)	74/87	41/61
PAC (349–392 aa)	95/95	75/95	89/97	CTT motif (710–719 aa)	90/100	60/70
19 (1129–1179 aa)	—	50/86	41/65	TPBD2 (815–953 aa)	67/81	50/66
***Es*** **CYCLE/BMAL**	***P. leniusculus*** **(** ***pl*** **BMAL1a)**	***D. melanogaster***	***M. musculus*** **(** ***m*** **BMAL1b)**	***Es*** **CRYPTOCHROME 1**	***D. nigrofasciatus***	***D. melanogaster***
Full-length protein	69/80	34/46	45/60	Full-length protein	56/69	50/63
HLH (84–135 aa)	100/100	78/90	74/86	DNA-photol. (7–176 aa)	55/67	54/69
PASA (150–217 aa)	94/97	73/89	75/91	FAD-bind. (220–498 aa)	65/78	54/66
PASB (340–406 aa)	86/94	51/80	57/76	CCE (482–533 aa)	40/59	33/39
PAC (413–456 aa)	82/93	68/85	51/80	CTT motif (517–526 aa)	60/70	50/50
BCTR (625–664 aa)	97/97	—	82/86			
***Es*** **PERIOD**	***N. norvegicus***	***D. melanogaster***	***M. musculus*** **(** ***m*** **PER2)**	***Es*** **CRYPTOCHROME 2**	***E. pulchra***	***M. musculus*** **(** ***m*** **CRY1)**
Full-length protein	52/63	23/36	21/36	Full-length protein	70/82	59/70
PASA (210–277 aa)	84/89	56/72	29/47	DNA-photol. (5–168 aa)	86/94	77/88
PASB (357–423 aa)	80/84	44/63	38/53	FAD-bind. (213–486 aa)	72/84	67/79
PAC (431–474 aa)	89/97	64/75	57/73	CC domain (471–493 aa)	78/86	59/81
DBT/CK1 (692–720 aa)	54/82	48/73	32/63			
Period-C (1013–1215 aa)	60/70	—	30/55			

*Alignment limited to the 286–298 aa region of *Es*TIM1.

### Positive loop components

We identified *Es*CLOCK (*Es*CLK) and *Es*CYC*LE*/BMAL (*Es*CYC/BMAL) as the putative positive elements of the first transcriptional and translational feedback loop in krill. Domains responsible for the interactions with the E-box (bHLH) and between CLOCK and CYCLE/BMAL (PAS-A, PAS-B, and PAC domains^34^), are highly conserved in *Es*CLK and *Es*CYC/BMAL (Table 1 and Fig. 1) suggesting the *Es*CLK:*Es*CYC/BMAL dimer formation in krill.

CLOCK C-terminal tails, downstream of the PAC domain, are generally less conserved even among closely related species. However, several organisms including crustaceans, insects, and vertebrates share glutamine-rich (Q-rich) regions that are essential for the *d*CLK transcriptional activity^35^. Two Q-rich regions (aa positions 708–730 and 899–1056) were identified (Fig. 1), composed of 83% and 69% Q residues respectively (Q percentages calculated according to Chang *et al*.^36^). A third region with a high frequency of Q residues (38%) was located at aa position 506–649. Krill CLOCK’s main Q-rich region is the second longest in length ever found with 109 Q residues within a 158 amino acids region.

In mouse, exon 19, and not the Q-rich region, plays a fundamental structural/regulatory role for the *m*CLOCK:*m*BMAL1 dimer function; the deletion of this region (*m*CLOCKΔ19) results in a loss of transactivation activity^37^. The sequence corresponding to the *m*CLOCK’s exon 19 is conserved in krill as well as in other crustaceans, insects, and vertebrates (Table 1 and Supplementary Figure 1A). Therefore, a similar structural/regulatory role might be proposed for *Es*CLK.

Recently, *m*CLOCK has been shown to possess a histone acetyltransferase activity^38^. However, due to the lack of the corresponding conserved domain, a similar role for *Es*CLK is unlikely.

**Figure 2:**
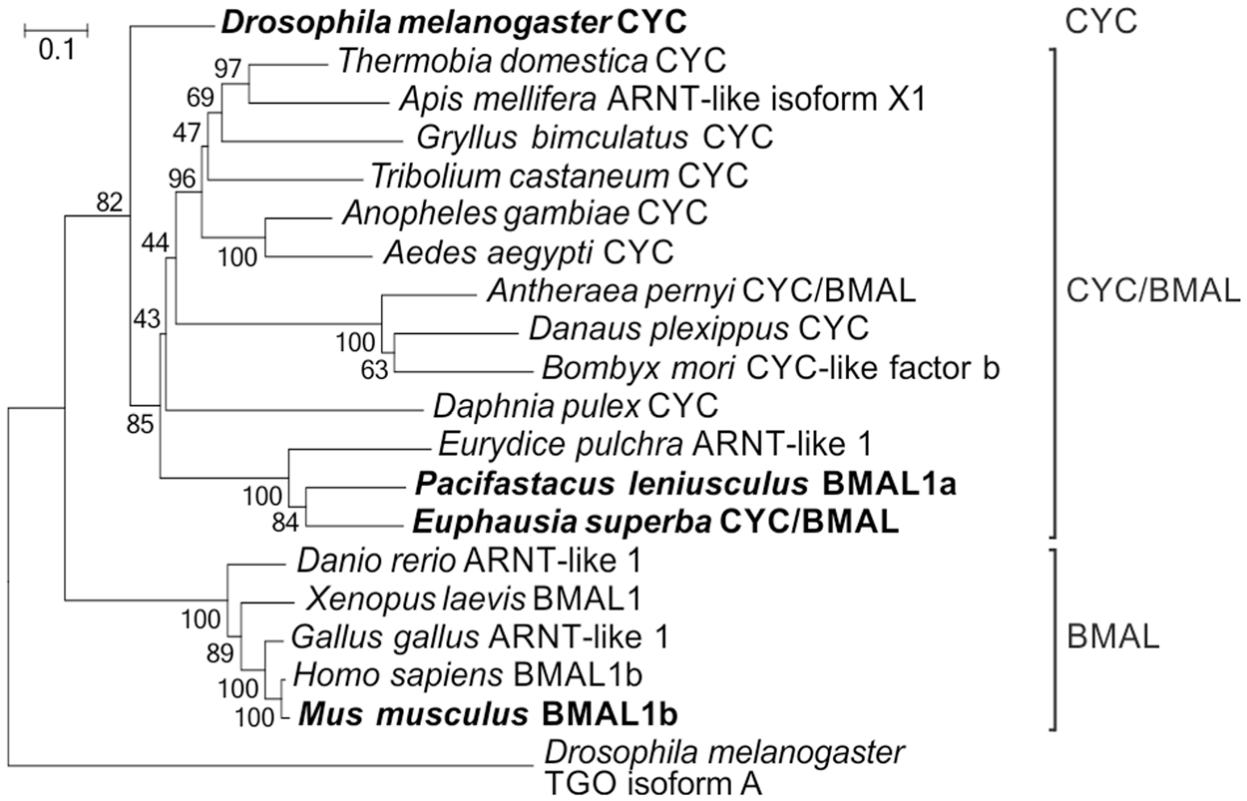
Phylogenetic relationships of the CYCLE/BMAL protein family. The *D. melanogaster*’s bHLH-PAS protein TANGO isoform A has been used as outgroup. Bootstrap confidence values based on 1,000 replicates are shown at nodes. Scale bar indicates amino acid substitutions per site. The most relevant orthologues are indicated in bold.

CYCLE/BMAL’s BCTR domain (Supplementary Figure 2) is essential for transactivation activity of the CLK:CYC/BMAL dimer in mammals^39^, in *E. pulchra* ^31^ and in *A. pernyi*, but has been lost during evolution of *d*CYC^40^. The highly conserved BCTR domain of *Es*CYC/BMAL unambiguously identifies the protein as a mammalian-like CYCLE (Figs 1, 2 and Table 1). Nuclear localization signals (NLS) and a nuclear export signals (NES) were identified at conserved positions close to the bHLH and PAS-A domains^41^ in both *Es*CLK and *Es*CYC/BMAL (Fig. 1 and Supplementary Table 2). It is likely that *Es*CLK:*Es*CYC/BMAL dimerization, via their PAS domains, could mask the localization signals, affecting the subcellular distribution of the dimer as suggested in mouse^41^.

### Negative loop components

We identified *Es*PER, *Es*TIM, and the previously reported *Es*CRY2^32^ as the putative negative elements of the first transcriptional and translational feedback loop in krill. The highly conserved *Es*PER’s N-terminal region consists of the heterodimerization domains (PAS-A, PAS-B and PAC/CLS), but not the bHLH domain identified in *Es*CLK and *Es*CYC/BMAL (Table 1 and Fig. 1). The lack of the DNA binding domain (bHLH) strengthens the annotation as a PERIOD protein that, unlike CLK and CYC, plays its role in the first transcriptional and translational feedback loop without a direct binding to DNA.

In *d*PER the CLK/CYC inhibitory domain (CCID) was mapped in the C-terminus at aa position 764–1034^36^. We have no evidence supporting the presence of a conserved inhibitory domain in *Es*PER or in PER proteins from other crustaceans (Fig. 1). This observation, as well as the conservation of the Period_C domain involved in the *m*PER2-*m*CRYs binding, suggests a regulatory/stabilizing role for *Es*PER, strengthening the hypothesis of a vertebrate-like negative loop in krill with *Es*CRY2 as the *Es*CLK:*Es*CYC/BMAL inhibitor. In mouse, two different binding models have been proposed for the interaction between *m*PER2’s C-terminal domain and the inhibitory cryptochromes *m*CRY1^42^ and *m*CRY2^43^, respectively. The higher homology to *m*CRY1, as well as the conservation of all the residues involved, suggest the *m*PER2:*m*CRY1 model as a reference for the *Es*PER-*Es*CRY2 interaction (Supplementary Figure 3). About half of the residues involved in the interaction are, instead, conserved in *Es*PER. Nevertheless, this resembles what has been reported for *D. plexippus* where the PER:CRY2 dimer formation has been validated^44^ as part of a TIM1:PER:CRY2 complex.

*Es*TIM1’s domains analysis identified a region, ranging from amino acid 583 to 958, that is highly conserved in insects and crustaceans (Table 1 and Fig. 1). Interestingly, this region is involved in the binding of the PER’s PAS-B domain in *Drosophila* ^45^ suggesting a similar role also in *E. superba*. Moreover, since the *d*PER cytoplasmic localization domain (CLD) in the PAC domain is supposed to be involved in defining the subcellular localization of the *d*PER:*d*TIM dimer through a direct interaction with *d*TIM^45^; its conservation in *E. superba* (Table 1, Supplementary Table 2, and Fig. 1), with respect to insects, crustaceans and vertebrates, further supports the idea of a *Es*PER:*Es*TIM1 dimer formation.

In *d*TIM, a CLD domain has been identified in the last 160 residues which are absent in *Es*TIM1 (Fig. 1) as well as in all the available crustacean TIM1 proteins (Supplementary Figure 4).

The coiled-coil (CC) domain and the C-terminal NLS of the inhibitory cryptochrome *m*CRY1 are essential for the nuclear localization of the *m*PER2:*m*CRY1 dimer^46^. In krill, the C-terminal NLS is not conserved (Fig. 1 and Supplementary Table 2) suggesting an increased relevance of the other *Es*CRY2 localization domains (CC domain and the N-terminal NLS).

Downstream the C-domain, *d*PER shows a small serine/glycine-rich sequence, followed by a long threonine/glycine repeat (26 TG repeats), that is involved in temperature compensation of the clock^47^. Krill PERIOD as well as other PER orthologues from crustaceans (in particular *E. pulchra* and *N. norvegicus*) show at the same position a region enriched in serine/glycine and serine/glutamine repeats but not the TG repeat (Supplementary Figure 5A). The function of these regions has not been defined yet, but their conservation might suggest an important biological role in crustaceans.

Sequence analysis of *Es*PER and *Es*TIM1 revealed regions involved in the interaction with several kinases. In particular, the PERIOD-DOUBLETIME binding domain (DBT/CK1), highly conserved among insects and crustaceans, was identified at aa position 695–721 (Fig. 1). The DBT/CK1 binding domain shows 48% identity and 73% similarity to the *D. melanogaster* corresponding sequences (Table 1). A serine-rich domain (SRD), containing seven predicted phosphorylation sites, has been identified in *d*TIM, mapping at aa position 260–292^48^. Deletion of this region affects period length and *d*TIM mobility suggesting that the SRD contains phosphorylation sites for CK2 and DBT^49^. Multi-alignment analysis (Supplementary Figure 4A) revealed that most of the TIM1 sequences available from insects and crustaceans share a well-conserved SRD region even longer than the *Drosophila* SDR (with 27–33 additional highly conserved amino acids just upstream the SRD core) increasing the number of sites that could be phosphorylated. *Es*TIM1 contains a 46 amino acids long SRD (253–298) containing 10 *in silico* predicted phosphorylation sites with a high level of homology to the SRD of insects (50% identity to *B. mori* and 46% to *D. plexippus*) and crustaceans (66% identity to *E. pulchra*, Table 1).

### Light entrainment

**Figure 3:**
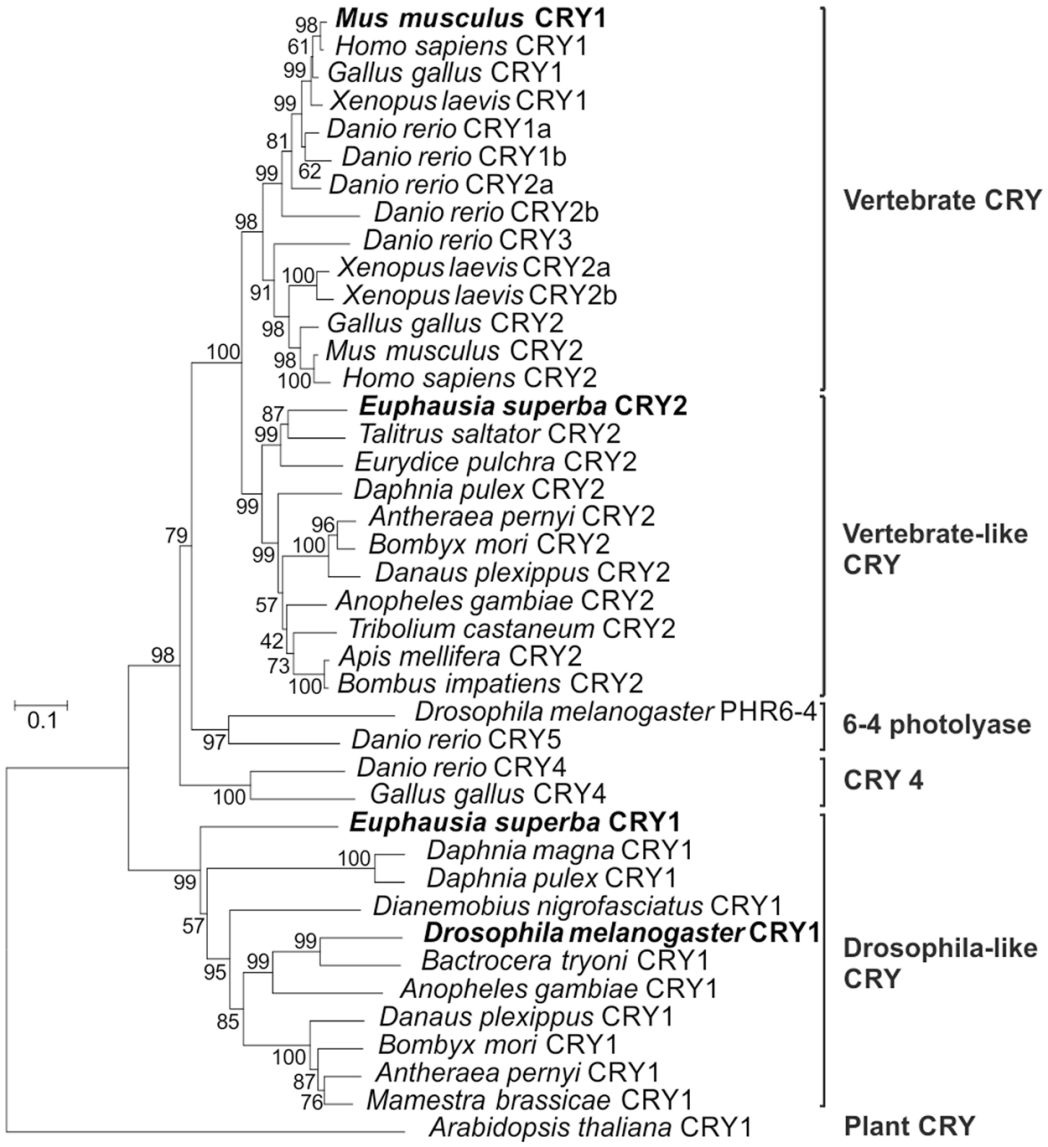
Phylogenetic relationships of CRYPTOCHROME protein family. The *A. thaliana*’s CRY has been used as outgroup. Bootstrap confidence values based on 1,000 replicates are shown at nodes. Scale bar indicates amino acid substitutions per site. The most relevant orthologues are indicated in bold.

Phylogenetic analysis unambiguously identified *Es*CRY1 as a putative light sensitive protein presumably involved in the photic resetting of the first transcriptional and translational feedback loop in krill (Fig. 3). The conservation of the N-terminal Photolyase-Homologous Region (DNA-photolyase binding domain and FAD binding domain; Table 1) and of all the three tryptophan residues (Trp triad) involved in the photoreduction of the flavin cofactor in the FAD binding domain in krill (Trp330, Trp384, and Trp407^50^) suggest a still functional *Es*CRY1 light-sensitive activity. Inside the CRY C-terminal Extension (CCE) region (aa 482–533), critical for nuclear/cytosol trafficking and protein-protein interactions, the putative *d*CRY’s TIM1 binding domain (CTT motif^51^) is moderately conserved (Table 1 and Supplementary Figure 6) but shows a positively charged arginine residue (R523) in the hydrophobic core motif that might be incompatible with the interaction. The *d*TIM’s putative *d*CRY1 binding site (CTT motif^51^), however, shows a higher level of conservation in *Es*TIM1 (Table 1 and Supplementary Figure 4A) supporting the hypothesis of a *Es*TIM1-*Es*CRY1 interaction in krill.

### *Es*CLK:*Es*CYC/BMAL dimer formation

**Figure 4:**
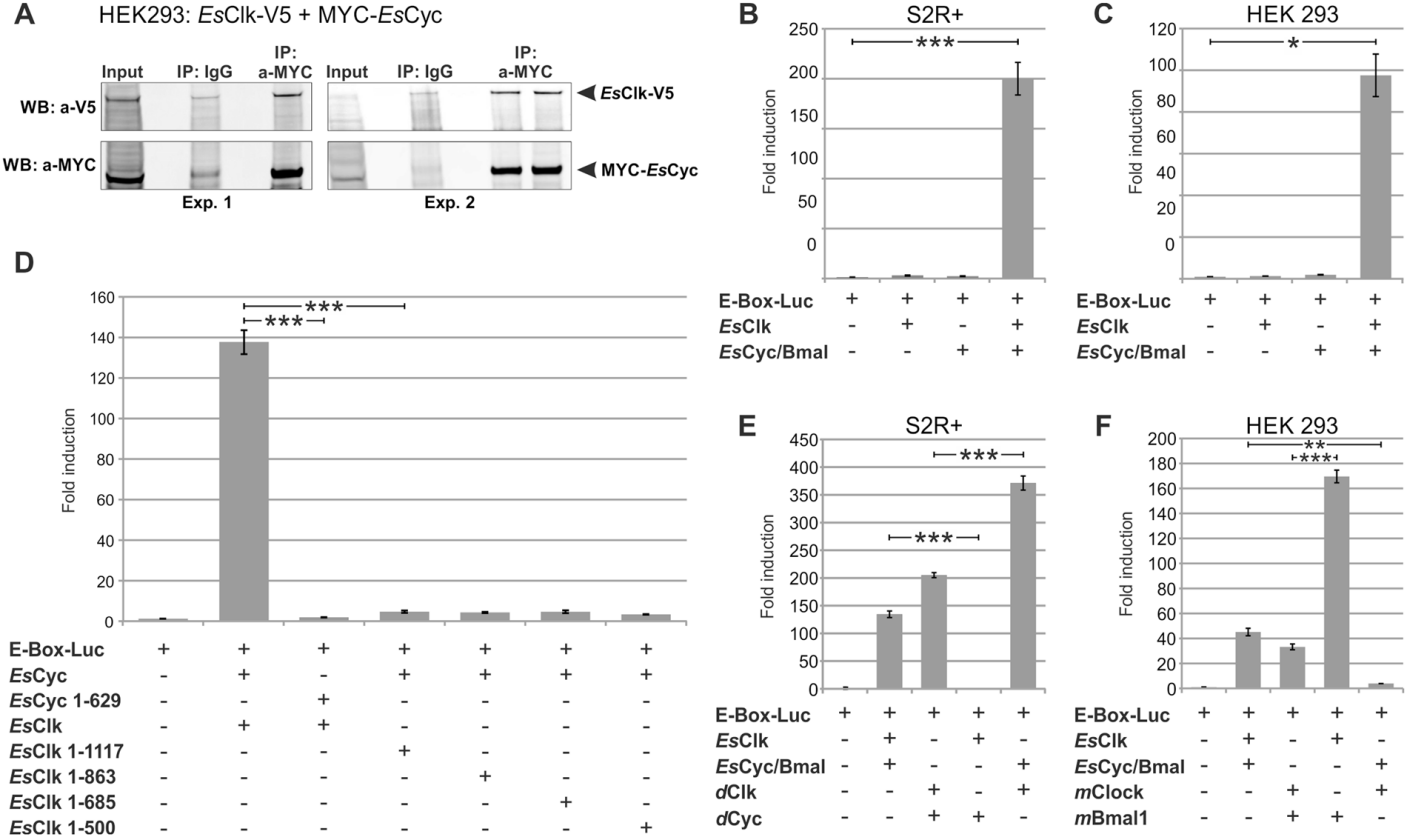
*Es*CLOCK and *Es*CYCLE/BMAL dimerize and activate transcription from the E-Box *in vitro*. (**A**) Co-immunoprecipitation of an epitope-tagged versions of *Es*CLK-V5 and MYC-*Es*CYC/BMAL co-expressed in HEK293 cells. Two experiments (Exp.) are reported showing that precipitates are enriched for *Es*Clock-V5. Membranes were probed with anti-MYC antibody to visualize pulldown efficiencies. For presentation purposes western blot images have been cropped (full-length blots are presented in Supplementary Figure S8A-C). (**B**,**C**) *Es*CLK and *Es*CYC/BMAL luciferase assay. *Es*CLK and *Es*CYC/BMAL - only as a heterodimer - activate the transcription of an E-box luciferase reporter in S2R + and HEK 293 cells, respectively. Cells were transfected with indicated constructs. Negative control set as 1. Data are represented as mean ± SD (n = 3 independent transfections). (**D**) Identification of conserved domains responsible for the transactivation activity of the *Es*CLK:*Es*CYC/BMAL by luciferase assay and their selective deletion. Data are represented as mean ± SD (n = 3 independent transfections). See Supplementary Figure 7 for a schematic representation of the constructs generated. (**E**,**F**) Interactions between *E. superba*’s positive clock elements with those of *D. melanogaster* and *M. musculus* evaluated by luciferase assay in S2R + and HEK 293 cells, respectively. Negative control set as 1. Data are represented as mean ± SD (n = 3 independent transfections). Student’s t-test Bonferroni-corrected p-values for all the experimental comparisons discussed were presented in Supplementary Table 3. Statistical significance of the most relevant comparisons were shown as *p < 0.05, **p < 0.01, and ***p < 0.005.

An evolutionary conserved interaction within the two most studied circadian clock models, *Drosophila* and mammals, is the heterodimerization of CLOCK and CYCLE (or BMAL) that act together as positive transcription factors. To test, whether *Es*CLK and *Es*CYC/BMAL can interact in living cells, we co-immunoprecipitated V5-tagged *Es*CLK and Myc-tagged *Es*CYC/BMAL from HEK293 cell extracts with both anti-Myc and anti-V5 antibodies (Fig. 4A). Both approaches revealed that these two proteins dimerize in mammalian cells.

Several features of the krill’s clock components showed similarities with those of the two circadian clock models. Therefore, in order to guarantee the most suitable molecular environment for the correct functioning of the krill clock components *in vitro*, we decided to perform our investigations in *Drosophila* S2R + cells as well as in mammalian HEK293 cells. Neither *Es*CLK or *Es*CYC/BMAL alone was able to activate the transcription of a E-box/luciferase reporter. Instead, the co-expression of *Es*CLK and *Es*CYC/BMAL resulted in a substantial increase in the luciferase signal in both *Drosophila* (Fig. 4B) and mammalian cells (Fig. 4C).

In mammals, *m*BMAL1 is the primary contributor to the *m*CLOCK:*m*BMAL1 dimer activity and the deletion of the BCTR domain suppresses the transactivation function. *m*CLOCK plays a structural/regulative role and the Q-rich regions and the exon 19 sequence corresponding region are responsible only for a transcriptional enhancing effect^37,39^. In *Drosophila*, however, *d*CYC lost the BCTR domain and *d*CLK became the primary contributor to the transactivation activity which is mediated by the Q-rich regions^40^. Truncated *Es*CYC/BMAL^1–629^, lacking the BCTR domain, abolishes the transactivation activity of the *Es*CLK:*Es*CYC/BMAL^1-629^ dimer consistent with the notion that the BCTR is the primary transactivation domain, as observed in mouse^39^ and *E. pulchra* ^31^ (Fig. 4D). Co-expression of full length *Es*CYC/BMAL and four mutant forms of *Es*CLK truncated just before the sequence corresponding to the murine exon 19 (*Es*CLK^1-1117^), the long Q-rich region (*Es*CLK^1-863^), the small Q-rich region (*Es*CLK^1-685^), and the domain with a high frequency of Q (*Es*CLK^1-500^) all dramatically diminished activity of the dimer as well. Deletion of the exon 19 corresponding sequence drastically reduced the transactivation activity, suggesting that the structural role of the corresponding aa sequence might be pivotal for the functioning of the *Es*CLK:*Es*CYC/BMAL dimer in krill. Deletion of the Q-rich regions did not further reduce the transcription of the E-box/luciferase reporter. There are at least two possible explanations for this result: 1) the *Es*CLK Q-rich regions might not possess the strong transactivation function observed in *Drosophila*; 2) deletion of the *Es*CLK C-terminal region, containing the exon 19 corresponding sequence, could impair correct folding and function of the Q-rich tail. The first hypothesis seems in accordance with our observation that in the presence of a complete and functioning *Es*CLK the deletion of the *Es*CYC/BMAL’s BCTR does not prevent loss of luciferase signal. Taken together these results suggest that full-length *Es*CLK and *Es*CYC/BMAL are both necessary for the induction of transcription from the E-box enhancer elements.

In order to understand whether the functioning of the positive feedback loop is more similar to the *Drosophila* or mammalian model, we investigated whether *Es*CLK and *Es*CYC/BMAL were able to replace the primary component of the CLK:CYC/BMAL dimer in *Drosophila* and in mammals, respectively. *Es*CLK was not able to replace *d*CLK in *Drosophila* cells (Fig. 4E) supporting the hypothesis that krill CLK’s Q-rich tail does not possess any transactivation activity. In addition, *Es*CYC/BMAL, when co-expressed along with *m*CLOCK in mammalian cells, cannot replace *m*BMAL1 resulting in a very low induction of the E-box/luciferase reporter (Fig. 4F). Presumably the secondary/enhancing role of mammalian CLOCK is not sufficient to support the dimer activity in krill suggesting an equal relevance of *Es*CLK and *Es*CYC/BMAL for the transactivation activity. Another possible explanation could be a structural or functional incompatibility of the clock components due to the high evolutionary distance between krill, *Drosophila*, and mammals. Nevertheless, in complementary experiments of co-expression of *d*CLK and *Es*CYC/BMAL in S2R + cells and *Es*CLK and *m*BMAL1 in HEK293 cells, the level of induction of luciferase reporter doubles compared to the respective positive controls (*d*CLK:*d*CYC and *m*CLK:*m*BMAL) suggesting not only a high compatibility between the components but also a synergy. The result obtained in *Drosophila* cells could be explained by the presence of two functioning transactivation domains in the dimer: the *Es*CYC/BMAL’s BCTR domain and the *d*CLK’s Q-rich regions that replaces the apparently inactive krill’s Q-rich region. For the results in mammalian cells there are two possible explanations. *Es*CLK’s exon 19 corresponding region, that become essential in krill, is able to dramatically increase the mammalian BCTR effect. Alternatively, *Es*CLK’s Q-rich regions evolved a transactivation activity as observed in *Drosophila*. However, since the previous experiments seem to weaken the hypothesis of an active role of the krill’s Q-rich for the activity of the *Es*CLK:*Es*CYC/BMAL dimer, the first hypothesis appears more likely. Together, these results strengthen our conclusion that both *Es*CLK and *Es*CYC/BMAL are pivotal for the *Es*CLK:*Es*CYC/BMAL dimer activity and, in particular, the role of *Es*CLK is more important than the simple transactivation/enhancing effect observed in mouse.

### *Es*CLK:*Es*CYC/BMAL inhibitors

**Figure 5:**
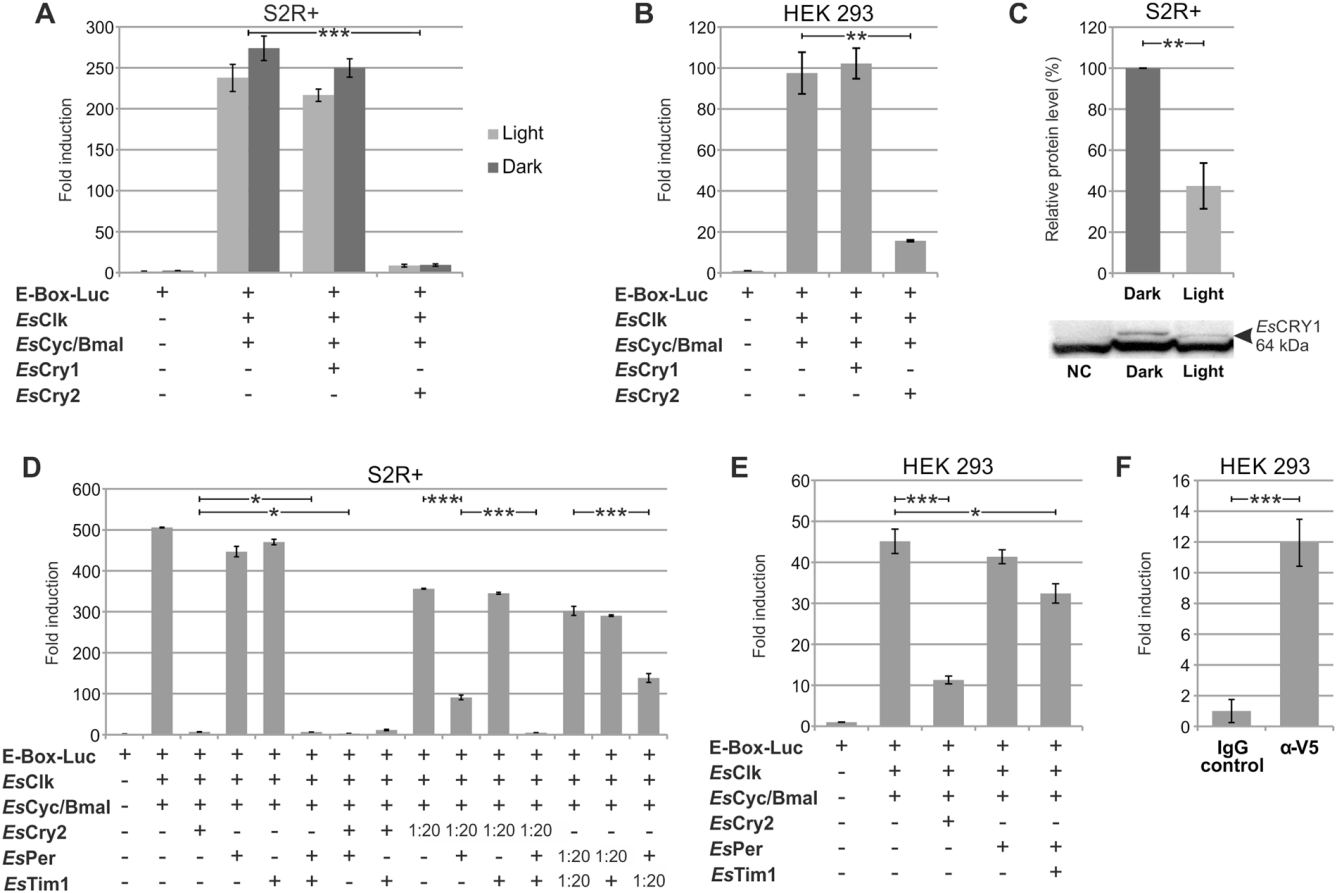
Functional characterization of the putative *Es*CLK:*Es*CYC/BMAL’s inhibitors. (**A**,**B**) *Es*CRY1 and *Es*CRY2 functional validation by luciferase assay in S2R + and HEK293 cells, respectively. Cells were transfected with indicated constructs. Negative control set as 1. Data are represented as mean ± SEM (n = 3 independent transfections). (**C**) Western blot and relative quantification of *Es*CRY1 protein in the dark and after a 8 hours light pulse in Drosophila cells. Data are represented as mean ± SD (n = 3 independent transfections). NC: negative control. (**D**,**E**) Comparison of the effectiveness of *Es*PER, *Es*TIM1, and *Es*CRY2 for inhibiting the transcription of the E-box/luciferase reporter mediated by the *Es*CLK:*Es*CYC/BMAL dimer in S2R + and HEK293 cells respectively. S2R + and HEK293 cells were transfected with the indicated constructs. Negative control set as 1. Data are represented as mean ± SD (n = 3 independent transfections). (**F**) Co-immonoprecipitation of *Es*CRY2 and *Es*CYC/BMAL quantified by luciferase assay. *Es*Cyc/Bmal C-terminally fused to luciferase (*Es*Cyc/Bmal-LUC) was co-immunoprecipitated with *Es*Cry2-V5 and anti-V5 antidody in HEK293 cells. Data are presented as mean ± SD (n = 3 independent transfections). Student’s t-test Bonferroni-corrected p-values for all the experimental comparisons discussed were presented in Supplementary Table 3. Statistical significance of the most relevant comparisons were shown as *p < 0.05, **p < 0.01, and ***p < 0.005.

The expression of *Es*CRY1 and *Es*CRY2 in S2R + and HEK293 cell lines along with *Es*CLK, *Es*CYC/BMAL, and the E-box/luciferase reporter, demonstrated that *Es*CRY2, but not *Es*CRY1, was able to inhibit the *Es*CLK:*Es*CYC/BMAL-mediated transcription in *Drosophila* cells (Fig. 5A) as well as in mammalian cells (Fig. 5B). Moreover, *Es*CRY2’s inhibitory power was unaffected by constant light or dark conditions in S2R + cells (Fig. 5A). On the other hand, western blot quantification after a 8 hours saturating light pulse showed a 60% decrease in the abundance of *Es*CRY1 in S2R + cells (Fig. 5C). Although the reduction in *Es*CRY1 levels was not as strong as in *Drosophila* (about 100% decrease), this result is comparable with the effects observed on butterfly’s CRY1 abundance after light treatment^19^. These results confirm the annotation of *Es*CRY2 as a vertebrate-like cryptochrome and *Es*CRY1 as a light sensitive protein (Fig. 3). In the light of these findings, we decided to take the *D. plexippus* butterfly’s molecular clock as a model to elucidate the functioning of the negative feedback loop in krill. Here, *Dp*TIM, *Dp*PER, and *Dp*CRY2 form a complex which promotes nuclear entry and stabilizes the *Dp*CRY2 mediated inhibition of *Dp*CLK:*Dp*CYC^52^. To test whether *Es*CRY2 is the krill’s primary inhibitor, we compared the effectiveness of *Es*PER, *Es*TIM1, and *Es*CRY2 as inhibitors of the transcriptional activation mediated by the *Es*CLK:*Es*CYC/BMAL dimer in S2R + (Fig. 5D) and HEK293 cells (Fig. 5E). *Es*PER and *Es*TIM1 showed a considerably weaker inhibitory power compared to *Es*CRY2. Moreover, we performed a co-immunoprecipitation of *Es*CYC/BMAL C-terminally fused to luciferase (*Es*Cyc/Bmal–LUC) with V5-tagged *Es*CRY2 and an anti-V5 antibody in HEK293 cells (Fig. 5F). The observed high inhibitory power and the direct interaction with *Es*CYC/BMAL suggests that *Es*CRY2 is the primary inhibitor of the *Es*CLK:*Es*CYC/BMAL dimer in krill.

Interestingly, the coexpression of *Es*PER and *Es*TIM1 (a dimerization domain is present in both proteins) resulted in a strong inhibition of the transactivation activity of the *Es*CLK:*Es*CYC/BMAL dimer in *Drosophila* cells (Fig. 5D). The inhibitory power of *Es*PER:*Es*TIM1 was stronger than observed for *Es*CRY2 alone, but slightly weaker compared to when *Es*PER and *Es*CRY2 were coexpressed in S2R + (Fig. 5D). We decided to test whether *Es*PER can modulate the inhibitory activity of *Es*CRY2 in S2R + cells line (Fig. 5D). Due to the strong effect of *Es*CRY2 on the *Es*CLK:*Es*CYC/BMAL-mediated transactivation activity we decreased the transfected amount of *Es*Cry2 by twenty times leading to a 80% reduction in its inhibitory power. *Es*PER alone resulted in a 10% reduction of the *Es*CLK:*Es*CYC/BMAL dimer activity; but its inhibitory power rises up to about 75% in the presence of even a low amount of *Es*CRY2. These results suggest a synergic rather than an additive contribution of *Es*PER and *Es*CRY2 on the heterodimer inhibitory activity. This hypothesis is supported by the presence of a CRY2 interaction domain in *Es*PER C-terminus (Fig. 1) as well as of a well-conserved PER binding residues in *Es*CRY2 sequence (Supplementary Figure 3A). Nevertheless, an *Es*PER:*Es*CRY2 dimer formation has not been detected in our co-immunoprecipitation experiments.

Then, we focused our investigations on the role of *Es*TIM1. The results obtained by the co-expression of *Es*TIM1 and a low amount of *Es*CRY2 (Fig. 5D) demonstrated a lack of synergic activity suggesting that the inhibitory activity observed was simply additive. Interestingly, according to our luciferase experiments with a low amount of *Es*CRY2, the co-expression of *Es*PER with *Es*TIM1 results in a inhibitory effect significantly higher than the expression of *Es*PER alone suggesting that the CRY2:PER:TIM1 complex should be regarded as the more effective inhibitor of the krill’s circadian clock as proposed for *D. plexippus*.

However, the high inhibitory power of the *Es*PER:*Es*TIM1 dimer cannot be ignored. In order to understand the specific role, we decreased the transfected amount of *Es*PER and *Es*TIM1 and tried to increase the inhibitory effect of the dimer by restoring the full amount of the two components one by one. The increased amount of *Es*TIM1 was not able to further affect the *Es*CLK:*Es*CYC/BMAL mediated transactivation activity suggesting a more stabilizing role; whereas, the amount of *Es*PER was directly proportional to the detected inhibitory effect. Despite the lack of the CLK/CYC inhibitory domain at the C-terminus of *Es*PER, these results are consistent with a prominent role of *Es*PER for the inhibitory activity of the *Es*PER:*Es*TIM1 dimer. Since the transfection of *Es*PER alone does not affect the *Es*CLK:*Es*CYC/BMAL-mediated transactivation activity, it seems likely that *Es*PER’s inhibitory effect is supported by the interaction with *Es*TIM1 that presumably stabilizes *Es*PER like in *Drosophila*.

### Temporal expression profiles

**Figure 6:**
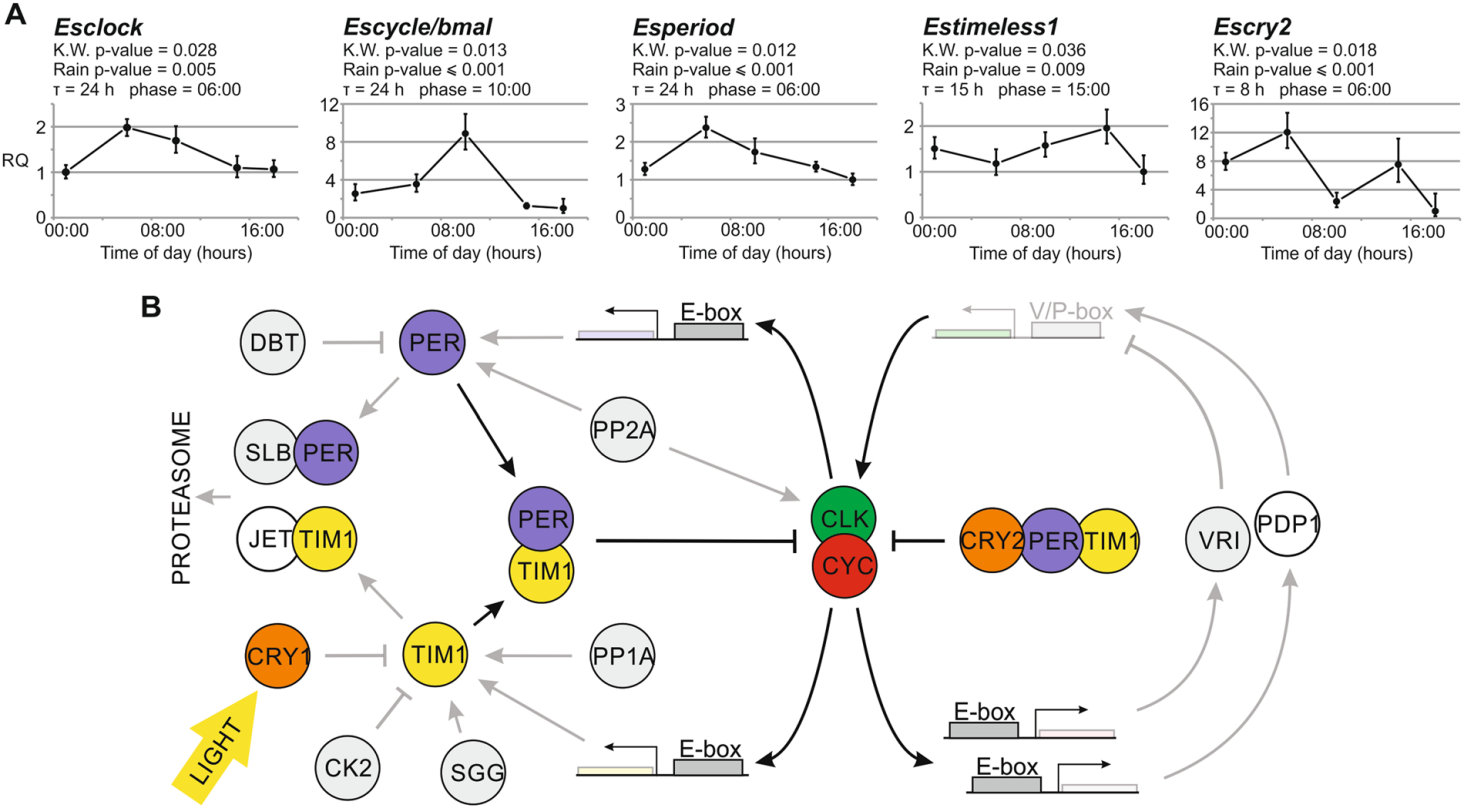
Putative functioning of the circadian clock machinery in *E. superba*. (**A**) Temporal patterns of expression of the five main circadian clock components (*Esclock*, *Escycle/bmal*, *Esperiod*, *Estimeless1*, and *Escry2*) in the eyestalks of krills sampled at 1:00, 6:00, 10:00, 15:00, and 18:00 during the Antarctic summer (almost 24 hours of light). Relative quantification (RQ) is represented as mean ± SD (n = 3 pools of 10 eyestalks each). Kruskal-Wallis p-value is reported, as well as adjusted p-value, period (τ) and phase of the oscillation estimated using RAIN algorithm. (**B**) A schematic model of the circadian clock in *E. superba*. The two main interlocked feedback loops are represented. The clock components identified in *E. superba* are colored; components sequenced but not functionally characterized are in grey (Supplementary Table 1); PDP1 and JET albeit only suggested by our data have been recently identified by Hunt *et al*.^68^.

In order to test whether the identified clock components show circadian oscillations at the transcriptional level, we examined the temporal expression profiles in krill eyestalks and brain sampled from nature during the Antarctic summer^32^. *Esclock*, *Escycle*/*bmal*, *Esperiod*, *Estimeless1*, and *Escryptochrome2* (Fig. 6A) were significantly differentially expressed around the 24 hours (Kruskal-Wallis p-value < 0.05). Albeit five-time points are not sufficient to provide a robust prediction of phase and periodicity, the RAIN analysis suggested daily rhythmic patterns of expression for the above-mentioned clock genes (adjusted p-value < 0.05). The comparison of daily expression profiles between positive and negative clock components do not show the typical antiphase trends observed in mammals and insects. However, unusual patterns of gene expression have already been described in crustaceans; for instance, in *E. pulchra* only *timeless1* showed significant oscillations in abundance around the 24 hours under DD conditions^31^, and in *Procambarus clarkii* PER, TIM, and CLK shared the same phase in the brain under LD conditions^53^.

## Discussion

Three different molecular clock models have been characterized in insects: in two of them only CRY1 (e.g. *D. melanogaster*) or CRY2 (e.g. *A. mellifera*) is present; whereas in the “ancestral clock” of *D. plexippus* both CRYs are expressed^19^. Here we characterized *Es*CRY2 as a vertebrate-like cryptochrome, a light-insensitive inhibitor of the *Es*CLK:*Es*CYC/BMAL dimer, and *Es*CRY1 as a *Drosophila-*like cryptochrome degraded by light. The high level of conservation of the two cryptochromes, in terms of sequence and specific functions, strongly suggests that the krill’s circadian clock could be regarded as representative of an ancestral circadian clock in crustaceans. Moreover, so far, both the cryptochromes have been identified only in *E. superba* and *D. pulex*, suggesting that the ancestral clock organization has been retained in few, even taxonomically distant, species.

Although we showed that *Es*CLK:*Es*CYC/BMAL dimer formation and activity are conserved in krill, the specific functional relevance of the domains contributing to transactivation activity does neither fully resemble the *Drosophila* nor the mammalian model. *Es*CLK evolved a large Q-rich tail like in *Drosophila* but the transactivation function of the *Es*CLK:*Es*CYC/BMAL dimer is still primarily mediated by the activity of the BCTR domain and the protein domain encoded by exon 19. Moreover, our results suggest that in krill, the region corresponding to exon 19 enhances the *Es*CLK:*Es*CYC/BMAL dimer transcriptional activity significantly more than the CLK homologous domain in vertebrates^39^.

*Es*CLK is characterized by two separated Q-rich regions but without a Q-stretch. In contrast, *D. pulex* CLK protein does not possess any glutamines repeats while in *M. rosenbergii* CLK the expansion of the long Q-stretch has almost completely replaced the exon 19 corresponding sequence, presumably impairing its function^27^ (Fig. 1). This high variability of the CLK glutamine-rich region extent among Crustacea suggests that this domain, but probably not its function, is conserved from an ancestral CLOCK protein. This situation resembles what is known in insects where several species have developed long Q-rich domains (e.g. *Anopheles gambiae* and *Aedes aegypti*); others have lost Q-repeats (e.g. *A. pernyi* ^36^ and *Lutzomyia longipalpis* ^54^); while in Brachycera (e.g. *D. melanogaster* and *Anastrepha fraterculus*) it has evolved into the primary transactivation domain of the CLK:CYC dimer, along with the loss of the CYC’s BCTR domain^55^. In contrast, the CLK’s exon 19 corresponding region and the CYC’s BCTR domain show a high level of conservation not only among Crustacea (*E. superba*, *D. pulex*, and *E. pulchra*) but also with insects and vertebrates (Supplementary Figure 1A and 2) suggesting a common ancestral CLK:CYC dimer in which these domains are equally responsible for the transactivation activity. Then, more recently the CLK protein has evolved a larger and more complex glutamine-rich region independently in the crustacean and insect lineages. The development of a functioning Q-rich transactivation domain could make the evolutionary older exon 19 and the BCTR domain dispensable or redundant, leading to their loss as it presumably happened in *M. rosembergii* ^27^ and in Brachycera^55^.

In contrast to the widespread conservation of TIM2, TIM1 is present in most of the insects (but not in *A. mellifera* or *Figulus rubripes*) and in all the available crustacean sequences, but it seems to be absent in vertebrates (e.g. *H. sapiens*), tunicata (e.g. *Ciona intestinalis*), and nematoda (e.g. *Caenorhabditis elegans*)^40^ suggesting that TIM1 evolved from a TIM2 duplication in the arthropod lineage^56^. In accordance with this hypothesis, TIM1 and TIM2 are both present in krill (Supplementary Figure 4B). However, a multi-alignment of TIM1 protein sequences revealed the absence of the C-terminal CLD domain (last 160 aa of *d*TIM^45^) from all available crustacean sequences (Supplementary Figure 4A). It has to be clarified whether the CLD function has been lost or replaced by another domain; in any case, this loss could have occurred in Arthropoda sometime after Crustacea diverged from Hexapoda.

The widespread conservation of the PER protein confirms its pivotal role in the ancestral molecular clock of vertebrates, insects, and crustaceans (Supplementary Figure 5B). In particular, the high level of conservation of PER-C domain (Table 1) and the absence of a CCID domain (Fig. 1) among crustaceans support the hypothesis of a mammalian-like role for the crustacean ancestral PER protein and consistent with the *D. plexippus* clock model, in which *Dp*CRY2 is the principal inhibitor of *Dp*CLK:*Dp*CYC/BMAL activity, whereas *Dp*PER only promotes *Dp*CRY2 nuclear entry. However, our luciferase experiments (Fig. 5D), suggested a dual role for *Es*PER as an enhancer of *Es*CRY2 activity, and as an active inhibitor stabilized by *Es*TIM1. Thus, the simple *D. plexippus* model, with PER stabilizing CRY2 and TIM1 stabilizing PER, does not fit with our findings in *E. superba*. In krill, inhibition of the *Es*CLK:*Es*CYC/BMAL dimer can be achieved not only through the *Es*CRY2:*Es*PER:*Es*TIM1 complex as in *D. plexippus*, but also by an *Es*PER:*Es*TIM1 dimer like in *Drosophila*. If the krill circadian clock is based on a dual negative feedback loop, a few questions arise: do these two inhibitory pathways share the same function? How are they spatially and temporally regulated? The development of two inhibitory pathways, likely characterized by different strength, period, and phase could represent an evolutionary strategy to increase the molecular clock plasticity to cope with the extreme seasonal changes which characterize the Southern Ocean environment.

The identification of the CTT motif in *Es*CRY1 and *Es*TIM1, as well as the light-sensitivity of *Es*CRY1, support the hypothesis of *Es*CRY1 as a circadian photoreceptor involved in the light induced entrainment of the molecular clock through the light-dependent degradation of *Es*TIM1. The conservation of such a molecular mechanism would suggest that light is a main *Zeitgeber* in krill and its circadian clock machinery is able to cope with the dramatic variability in annual day length. The oscillatory pattern of expression of the main molecular clock components (Fig. 6A) as well as the rhythmic diurnal expression profiles of hundreds of krill’s transcripts during the Antarctic summer^10^, represent an example of how evolution increased the plasticity of the temporal synchronization mechanism either by switching to alternative environmental cues or by compensating the lack of robust and stable *Zeitgebers* ^57,58^.

The complexity of the recently described krill’s photoreception system^11^, including 8 opsins characterized by light sensitivity ranging from long to medium wavelengths, suggests that not only the blue component of light, but the complete spectral composition could participate in the synchronization of the endogenous clock. Entrainment through retinal photoreceptors, observed in insects^59,60^, has also been demonstrated to be pivotal for two high latitude birds experiencing long periods of almost continuous light^61^.

This study sheds light on the molecular architecture and functioning of the krill’s circadian clock machinery (Fig. 6B) which has been predicted by several previous studies. The functional dissection of the first transcriptional and translational feedback loop reveals that krill possess a peculiar example of ancestral clock based on gears shared by vertebrates (vertebrate-like CYC, CRY2, and PER functioning) and insects (TIM duplication, and CRY1 role in photic entrainment). The *Es*CLK:*Es*CYC/BMAL dimer induces the transcription of genes under the control of E-box enhancer elements; whereas *Es*TIM1, *Es*PER, and *Es*CRY2 inhibit the *Es*CLK:*Es*CYC/BMAL mediated transcription. Moreover, phylogenetic analyses suggest that the CLOCK’s poly-Q expansion and the differential loss of *cryptochrome* in crustaceans follow models that have also been proposed in insects. Furthermore, our model proposes two main novelties that presumably take part in an evolutionary strategy to cope with polar environment’s challenges: the equal relevance of *Es*CLK and *Es*CYC/BMAL for the activity of the dimer and the dual inhibitory pathway (*Es*CRY2:*Es*PER:*Es*TIM1 and *Es*PER:*Es*TIM1). Finally, the photoreceptor *Es*CRY1, upon light exposure, could be involved in the *Es*TIM1 degradation, allowing the photic entrainment of the central oscillator. Despite the strong variability in annual day length that characterizes the high latitude regions, the conservation of this synchronization mechanism suggests the persisting pivotal role of light as a *Zeitgeiber* in krill.

## Materials and Methods

### Cloning of transcripts encoding canonical clock genes

Transcriptome mining was performed using BLAST + 2.6 software (NCBI, ftp://ftp.ncbi.nlm.nih.gov/blast/executables/blast+) and the first release of the *Euphausia superba* transcriptome database^33^. Total RNA was extracted with TRIzol (Invitrogen) from frozen heads sampled in 2004^32^. cDNA template was synthetized using SuperScript II Reverse Transcriptase (Thermo Fisher) with random hexamers (Thermo Fischer). Primers for PCR validation and 5′/3′ RACE were designed with the on-line software Primer3 version 4.0.0 (http://bioinfo.ut.ee/primer3). Complete coding sequences were cloned using the StrataClone Blunt PCR Cloning Kit (Clontech) and sequenced at BMR Genomics (Padova, Italy). Complete coding sequences were isolated with SMARTer RACE 5′/3′ kit (Clontech).

### Sequence analysis

Accession numbers for the protein sequences included in the analyses are reported in Supplementary Table 4. Phylogenetic trees were constructed with MUSCLE alignment tool and Neighbour‐Joining (NJ) method (Dayhoff substitution matrix and pairwise deletion) as implemented in MEGA 6.06 (http://www.megasoftware.net/). Protein sequences were aligned with Clustal Omega v1.2.4 (http://www.ebi.ac.uk/Tools/msa/clustalo). Protein sequences were colored using Jalview v2.10.1 (http://www.jalview.org) according to the default CLUSTALX conversion. We used: EMBL SMART version 7 (http://smart.embl.de) to detect PFAM domains and motifs of clock proteins; NLS Mapper (http://nls-mapper.iab.keio.ac.jp) for nuclear localisation signal prediction (cut-off = 5); NetNES version 1.1 (http://www.cbs.dtu.dk/services/NetNES) as well as consensus sequences for identifying nuclear export signals; and NetPhos 3.1 Server (http://www.cbs.dtu.dk/services/NetPhos/) to predict phosphorylation sites. The identity and similarity between proteins and domains were calculated with EMBOSS Pairwise Alignment Algorithms (EMBL‐EBI, http://www.ebi.ac.uk/Tools/emboss/).

### Constructs and S2 cells transcriptional activation assay

Sequences were cloned into S2 expression vectors pAC5.1/V5‐His A (Thermo Fisher) or pAc5-STABLE 2-neo (Addgene; gift from Rosa Barrio & James Sutherland^62^) with the In-Fusion HD cloning kit (Clontech) as described in Supplementary Table 5. The *Drosophila* E‐box luciferase reporter construct pGL3 4E‐hs‐luc consists of four *dper* E-box fused with a *hsp70* promoter upstream luciferase reporter^34^; kindly provided by Charalambos Kyriacou, University of Leicester, UK). The transfected amount of each constructs was calculated for a 1:1 molar ratio to 50 ng of pEsClk. The total amount of DNA was normalized using the empty Ac5-STABLE 2-neo vector and brought to 1 μg with an empty mammals pEt-28b (+) vector (Novagen). *Drosophila* S2R + cells (Invitrogen) were maintained at 25 °C in Schneider’s *Drosophila* medium (Thermo Scientific). Transfections were performed using Cellfectin reagent (Invitrogen). Transfection efficiency was assayed by GFP signal (expressed by the empty Ac5-STABLE 2-neo vector). After 48 h, cells were processed according to the Dual Luciferase Reporter Assay Kit (Promega). Luciferase activity was measured using a DLReady Luminometer TD20/20 (Turner Designs) and normalized with pCopia‐Renilla activity (Addgene; gift from Philip Beachy^63^). A negative control transfection (pGL3 4E‐hs‐luc, pCopia‐Renilla and empty Ac5-STABLE 2-neo) was used to establish the baseline reporter signal (set as 1). At least three independent transfections were performed for each assay. Comparisons’ significance was evaluated by t-test (2 tails; unequal variances; Bonferroni adjustments for multiple comparisons; adjusted p-value < 0.05).

### Constructs and HEK293 cell transcription assays

HEK293 cells were transfected with an artificial 6E-box luciferase reporter (pGL3, Promega) and krill or murine clock sequences in pDEST26 backbone (Invitrogen) using Lipofectamine 2000 (Invitrogen). Equal DNA amounts in transfections were ensured by adding lacZ DNA in the corresponding vector backbone. Co-transactivation assays were performed as previously described^37^. After 48 hours signal detection was performed with the Dual-Luciferase Reporter Assay (Promega) using the Orion II Luminometer plate reader (Berthold Detection Systems). Normalization was performed to Renilla-luciferase signals. Experiments were performed at least three times with similar results.

### Co-immunoprecipitation experiments

HEK293 cells were transfected with constructs expressing epitope or luciferase tagged krill proteins using V5: pLenti6 (Invitrogen); MYC: pc-myc-CMV-D12^64^; luciferase: pLenti6 (Invitrogen). Cells were harvested 48 hours after transfection in co-IP buffer (20 mM Tris-HCl at pH 8.0; 140 mM NaCl; 1.5 mM MgCl_2_; 1 mM TCEP; 1% Triton-X-100; 10% glycerin; 1X protease inhibitor cocktail, Sigma). 500 μg of total protein or 1 million counts per second (for luciferase containing lysates) were subjected to immunoprecipitation. Immunoprecipitation was performed with 2 μg of an anti-MYC (NB600-335, Novous Biologicals) or anti-V5 (R960-25, Invitrogen) antibody and G PLUS-agarose beads (sc-2002, Santa Cruz). As controls served isoform specific ideotypic antibodies (PP500P, Acris Antibodies or sc-2025, Santa Cruz Biotechnology). For Western blot analysis, beads were washed three times in washing buffer (20 mM Tris-HCl at pH 8.0; 150 mM NaCl; 0.5% Igepal CA-630). Proteins on beads were denatured by boiling in SDS-loading buffer (Invitrogen). Separation was performed by SDS-PAGE with 4%-12% Bis-Tris gels (Invitrogen). Proteins were transferred to nitrocellulose membrane using a wet tank transfer system (Biorad). Membranes were incubated with an anti-MYC or the anti-V5 antibody and probed HRP-conjugated secondary antibodies (Santa Cruz). Beads luciferase activity was measured in the Beta Scout Tester (PerkinElmer) as for measurements of luciferase activity in precipitates.

### *Es*CRY1 photosensitivity assay

S2R + cell were transfected with 300 ng of p*Es*Cry1. Culture plates were placed under fluorescent white lighting (7.3 klux) at 25 °C for 8 hours. Dark control plates were wrapped in aluminium foil and incubated next to the light-treated plates. Proteins were: extracted in TritonX-100 lysis buffer (20 mM Hepes pH 7.5; 100 mM KCl; 2.5 mM EDTA pH 8.0; 5% glycerol; 0.5% TritonX-100; 1 mM DTT; 1X Complete Protease Inhibitor Cocktail-Roche); separated by SDS-PAGE in a NuPAGE 4–12% Bis-Tris Protein Gels (Thermo Fisher); and transferred to a nitrocellulose membrane (Bio-Rad) by routine methods using a wet blottingsystem (Thermo Fisher). Western Blot membranes were incubated with monoclonal anti-HA antibody (Sigma) and then Anti-Mouse IgG-Peroxidase antibody (Sigma). Chemioluminescence reaction was performed with fresh made ECL buffer. Protein bands were visualized using Amersham Hyperfilm ECL (GE Healthcare). Relative abundance of *Es*CRY1 was normalized to the total protein amount per lane evaluated by Ponceau S staining^65^ (full-length blot and Ponceau S staining are shown in Supplementary Figure S8D). For quantification of the immunodetected signals, each film was analysed with Image J software (http://rsb.info.nih.gov/ij).

### Quantitative real-time PCR

1 μg of total RNA from a 10 eyestalks pool^32^ was used to perform independent cDNA syntheses for each time point (01:00, 06:00, 10:00, 15:00, and 18:00 h^32^). To avoid genomic DNA contamination, samples were treated with DNase I (Qiagen, Gaithersburg, MD, USA). Retrotranscription was performed using random hexamers (Thermo Fisher) and SuperScript II reverse transcriptase (Thermo Fisher). Three biological replicates were analysed. One μl aliquot of 1:100 diluted cDNA was PCR amplified in 10 μl volume using the GoTaq qPCR Master Mix (Promega). Gene-specific primers (Supplementary Table 6) were designed using the on-line software Primer3 version 4.0.0 (http://bioinfo.ut.ee/primer3). A dissociation curve was used to confirm the specificity of the amplicon. We verified primers’ efficiency by drawing standard curves for target genes and the spike. Amplifications were performed in triplicate in a 7500 Real-Time PCR System (Applied Biosystems). Since none of the previously used reference genes in krill^10,11,32^ proved to be a good housekeeping gene for these specific samples (isolated eyestalks from krills sampled from nature), results were normalized to an external spike-in synthetic oligorinucleotide added before the retro-transcription step at a final concentration of 20 pg/µl (Supplementary Table 6). The 2^−ΔΔCt^ method^66^ was used to calculate the relative expression ratio (RQ). Expression profile’s significance was evaluated by non-parametric Kruskal-Wallis test (p-value < 0.05; 4 degrees of freedom) whereas the estimated periodicity and phase of oscillation were obtained by RAIN algorithm^67^ (adjusted p-value < 0.05).

### Data availability

Cloned sequences are deposited in GenBank (IDs listed in Supplemental Table 1; https://www.ncbi.nlm.nih.gov/genbank).

## Electronic supplementary material

Supplementary Information
