# Supplementary material for: Functional characterization of the circadian clock in the Antarctic krill, Euphausia superba
Source: Sci Rep. 2017 Dec 18;7:17742. doi: 10.1038/s41598-017-18009-2 (PMC5735174; doi:10.1038/s41598-017-18009-2)
Supplement: Supplementary file 1 — Supplementary Information [file 41598_2017_18009_MOESM1_ESM.pdf]

Functional characterization of the circadian clock in the Antarctic krill, *Euphausia superba*

Alberto Biscontin[1,2], Thomas Wallach[1], Gabriele Sales[2], Astrid Grudziecki[1], Leonard Janke[1], Elena Sartori[2], Cristiano Bertolucci[3], Gabriella Mazzotta[2], Cristiano De Pittà[2], Bettina Meyer[4,5,6], Achim Kramer[1,*], Rodolfo Costa[2,*]

[1]Charité-Universitätsmedizin Berlin, Laboratory of Chronobiology, D-10117 Berlin, Germany; [2]University of Padova, Department of Biology, 35121 Padova, Italy; [3]University of Ferrara, Department of Life Sciences and Biotechnology, 44121 Ferrara, Italy; [4]Alfred Wegener Polar Biological Oceanography, 27570 Bremerhaven, Germany; [5]Carl von Ossietzky University of Oldenburg, Institute for Chemistry and Biology of the Marine Environment, 26129 Oldenburg, Germany; [6]Helmholtz Institute for Functional Marine Biodiversity Oldenburg (HIFMB), 26129 Oldenburg, Germany

[*]Corresponding Authors. E-mails: rodolfo.costa@unipd.it; achim.kramer@charite.de

Keywords: Antarctic krill; *Euphausia superba*; circadian clock; clock genes

SUPPLEMENTARY FIGURES

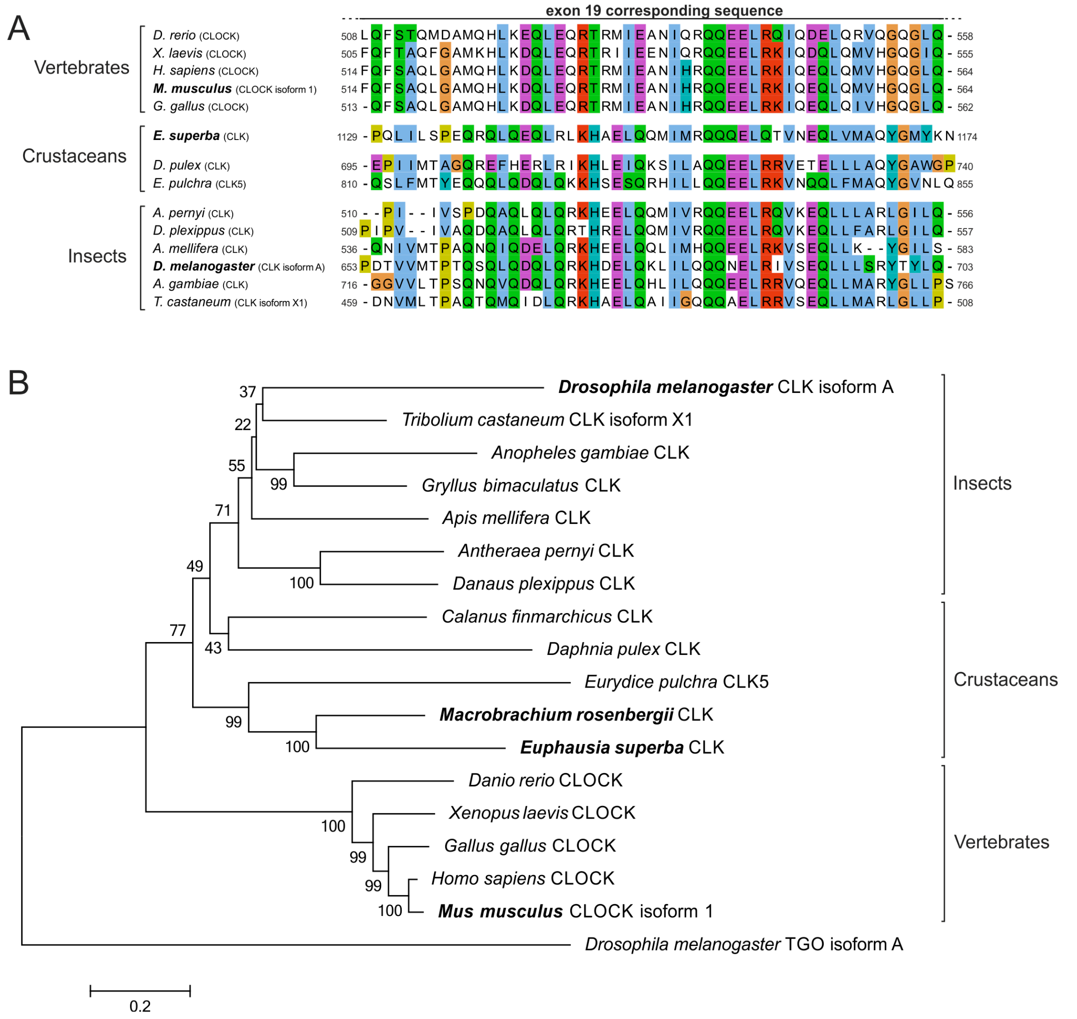

Fig. S1. *Es*CLOCK. (A) Multiple alignment of the CLOCK sequences corresponding to the *m*CLOCK's exon 19. (B) Phylogenetic relationships of the CLOCK protein family. *Es*CLK clusters with other crustaceans orthologues. The *D. melanogaster*'s bHLH-PAS protein TANGO isoform A has been used as outgroup. Bootstrap confidence values based on 1,000 replicates are shown at nodes. Scale bar indicates amino acid substitutions per site. The most relevant orthologues are indicated in bold.

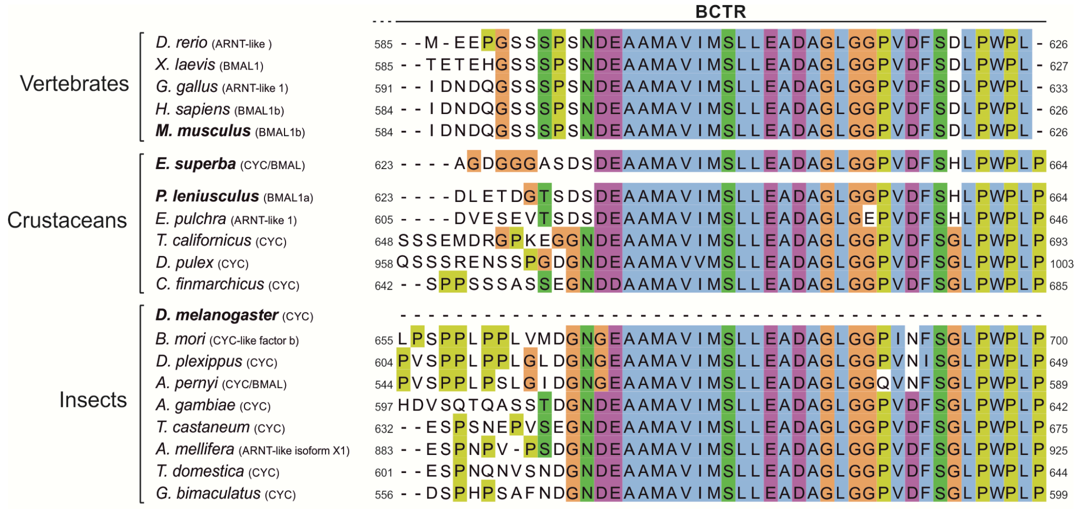

Fig. S2. *Es*CYCLE/BMAL. Multiple alignment of the CYC/BMAL sequences corresponding to the *m*BMAL1's BCTR domain.

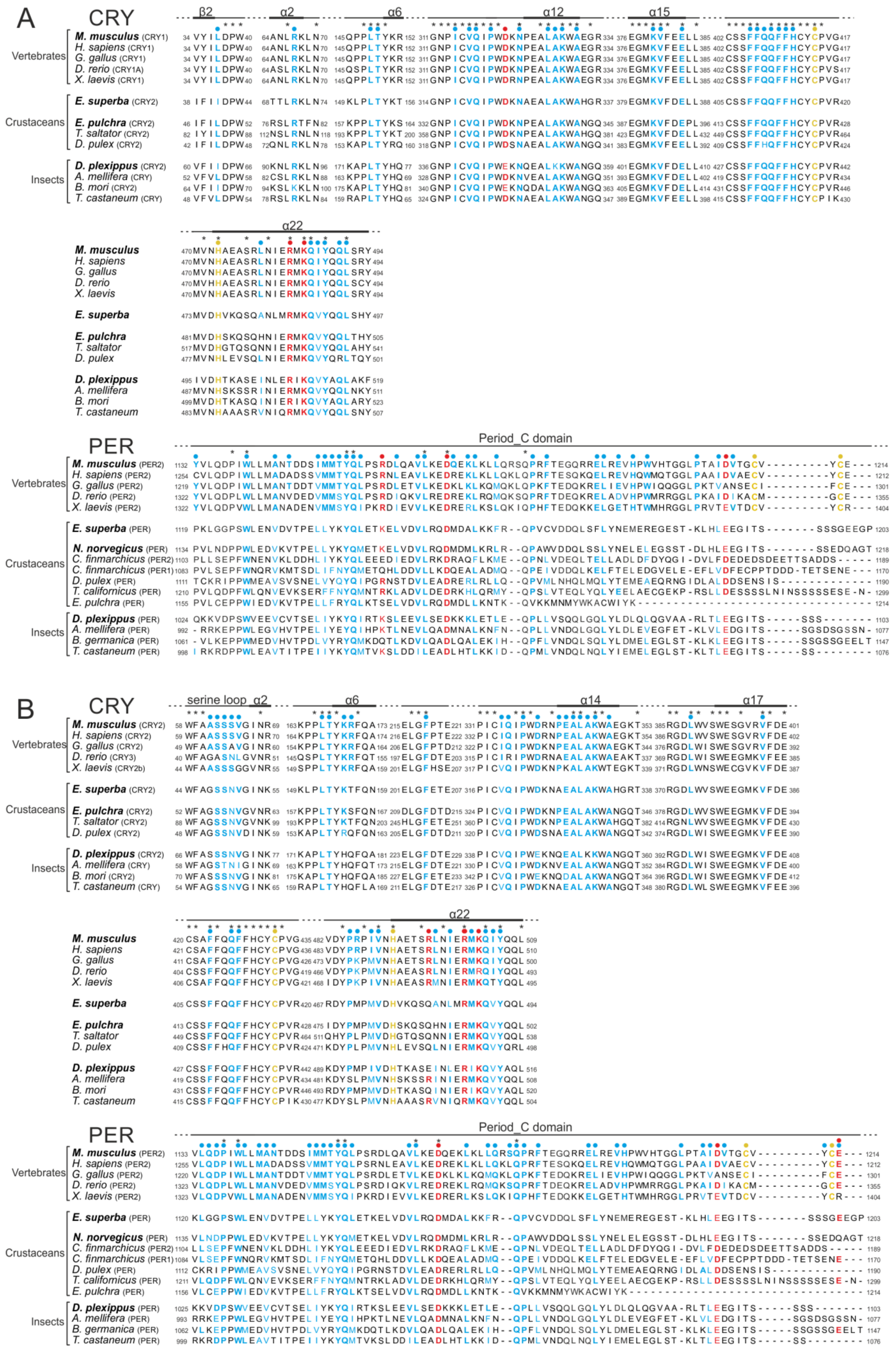

Fig. S3. Multiple sequence alignment of domains responsible for the PER/CRYs interaction in mouse according to the *m*PER2:*m*CRY1 (A) and *m*PER2:*m*CRY2 (B) models, respectively. (A) *m*PER2:*m*CRY1 interaction model (7): 29/29 residues involved in low energy interactions, 3/3 residues involved in salt bridges, and 2/2 residues involved in zinc finger are conserved in *Es*CRY2; 15/28 residues involved in low energy interactions, 3/3 residues involved in salt bridges, and 0/2 residues involved in zinc finger are conserved in *Es*PER. (B) *m*PER2:*m*CRY2 interaction model (8): 32/36 residues involved in low energy interactions, 2/3 residues involved in salt bridges, and 2/2 residues involved in zinc finger are conserved in *Es*CRY2; 18/34 residues involved in low energy interactions, 3/3 residues involved in salt bridges, and 0/2 residues involved in zinc finger are conserved in *Es*PER. Coloured residues represent the conserved amino acids (identity in bold font style) involved in low energy interactions (blue), salt bridges (red), and zinc fingers (yellow). * = the residue is conserved among all the sequences aligned; α = α-helix position in *m*CRY sequence.

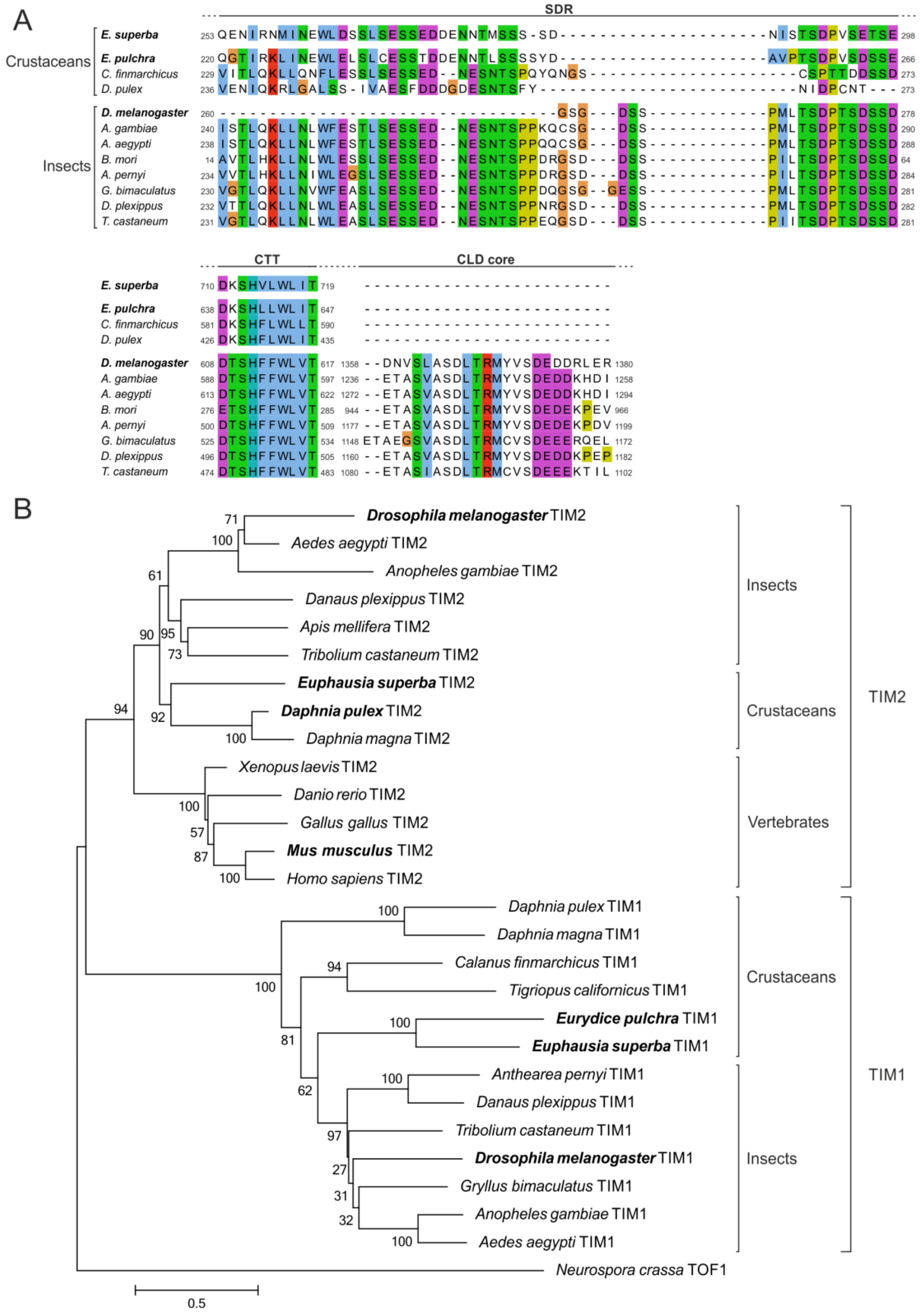

Fig. S4. *Es*TIMELESS1. (A) Multiple sequence alignment of serine-rich domain, putative *Es*CRY1 binding site (CTT motif), and the conserved region of the C-terminal cytoplasmic localization domain (CLD core). (B) Phylogenetic relationships of the TIMELESS protein family that confirm the annotations of *Es*TIM1 and *Es*TIM2 as a TIMELESS and TIMEOUT proteins, respectively. The *N. crassa* timeless domain-containing protein TGO1 has been used as outgroup. Bootstrap confidence values based on 1,000 replicates are shown at nodes. Scale bar indicates amino acid substitutions per site. The most relevant orthologues are indicated in bold

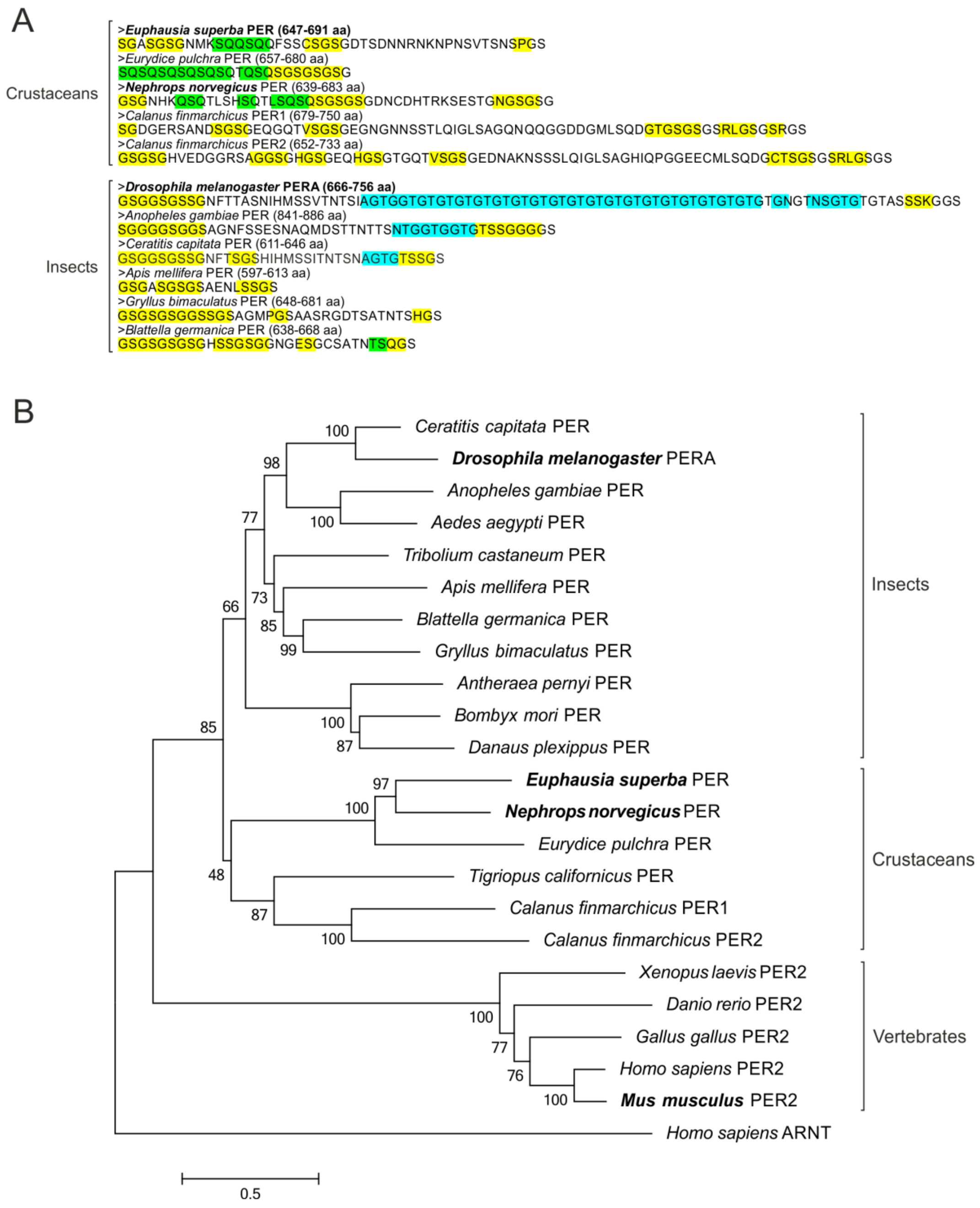

Fig. S5. *Es*PERIOD. (A) Visual comparison between T/G (blue), S/G (yellow), and S/Q (green) repeats found in relevant crustaceans and insects. (B) Phylogenetic relationships of the PERIOD protein family. *Es*PER clusters with other crustaceans orthologues. The *H. sapiens*'s bHLH-PAS protein ARNT has been used as outgroup. Bootstrap confidence values based on 1,000 replicates are shown at nodes. Scale bar indicates amino acid substitutions per site. The most relevant orthologues are indicated in bold

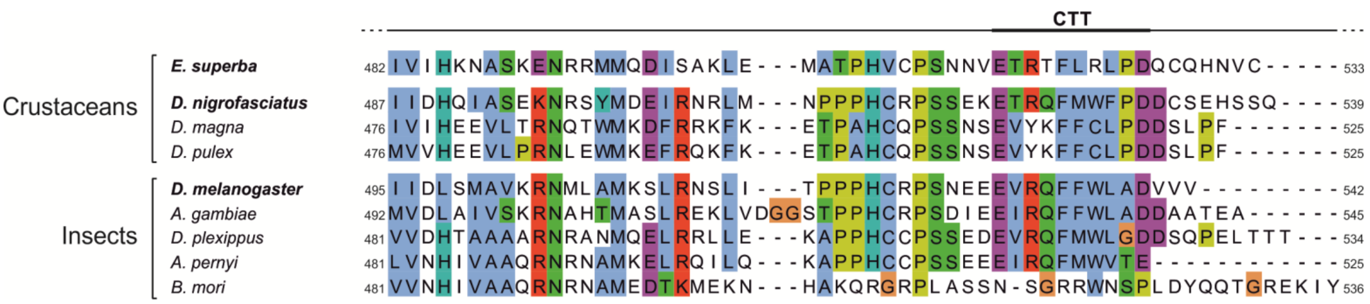

Fig. S6. *Es*CRY1. Multiple sequence alignment of CRY1's putative *Es*TIM1/*Es*CRY1 binding site (CTT motif) inside the CCE domain.

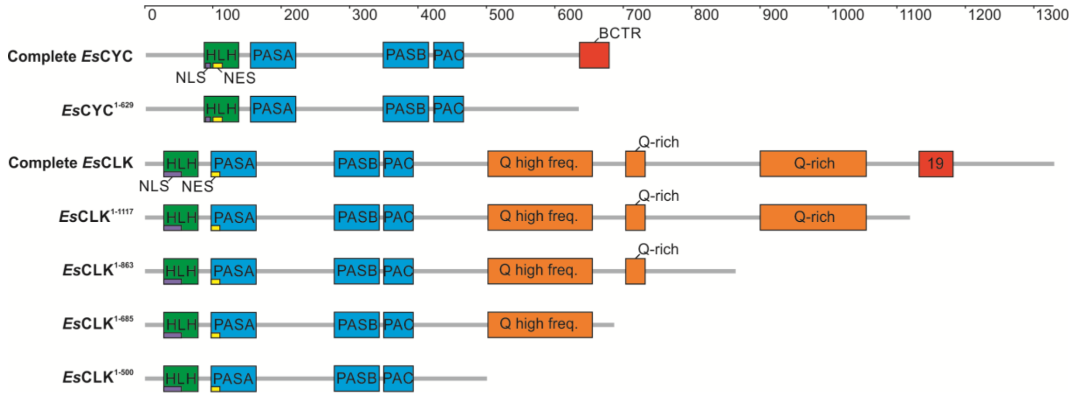

Fig. S7. Schematic representation of the constructs used in the Fig. 3D's experiment.

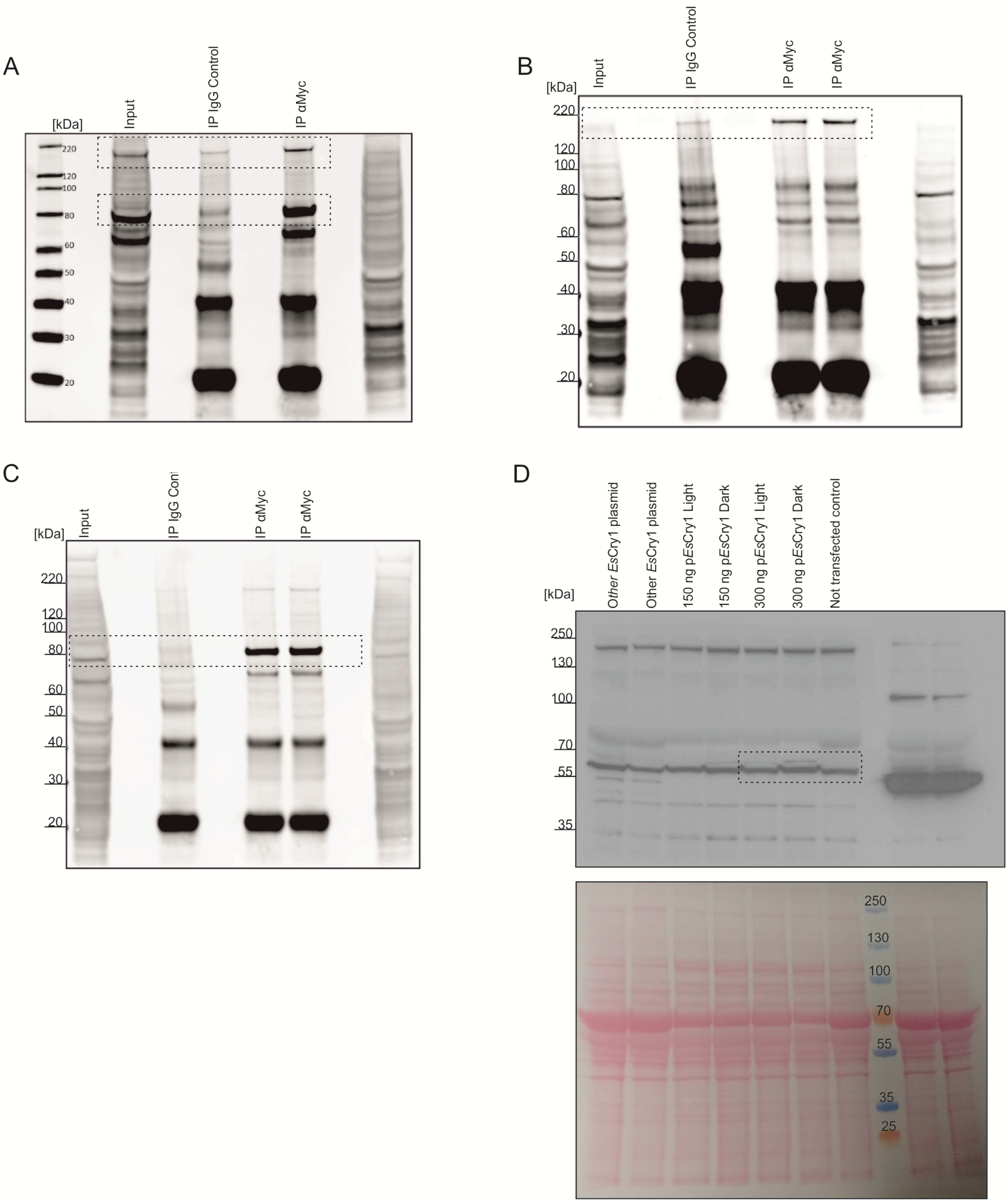

Fig. S8. Full-length western blot images. (A) Co-immunoprecipitation of *Es*CLK-V5 and MYC-*Es*CYC/BMAL co-expressed in HEK293 cells; experiment 1 αV5+αMyc, (B) experiment 2 αV5, and (C) experiment 2 αMyc (digital imaging). (D) Western blot of *Es*CRY1 protein after a 8 hours light pulse in *Drosophila* cells (film exposure) and Ponceau S staining used for total protein normalization (digital imaging). Dashed line rectangles mark bands displayed in the main text figures.

SUPPLEMENTARY TABLES

	Contig resulted from krill database mining		Transcript reconstruction by molecular cloning approach		Deduced protein information		tblastx search statistics		Sequence status
	Accession number	Length (nt)	GenBank accession number	Length (nt)	Protein length (aa)	Protein type	Score	E-value	
**CLOCK**	ESS034514	1659	KY888674	4652	1329	F	650	0	Confirmed
**CYCLE/BMAL**	ESS133965	303	KY922996	2767	664	F	911	0	Confirmed
**PERIOD**	ESS133963	2339	KY922997	4127	1261	F	1117	0	Confirmed
**TIMELESS 1**	ESS040526	4047	KY922998	5300	1311	F	1094	0	Confirmed
**CRYPTOCHROME 1**	ESS023688	1819	KY922999	1844	533	F	662	0	Confirmed
**DOUBLETIME**	ESS096455	1694	KY923000	3342	345	F	667	0	Validated
**SHAGGY**	ESS074789	1350	KY923001	1966	415	F	666	0	Validated
**VRILLE**	ESS123359	1509	KY923002	2224	488	F	233	5e-69	Validated
**SLIMB**	ESS133964	1350	KY923003	1457	449	C	764	0	Validated
**CASEIN KINASE 2α**	ESS071625	1722	-	-	350	F	629	0	Validated
**CASEIN KINASE 2β**	ESS051147	805	-	-	244	N	422	3e-148	Validated
**PROTEIN PHOSPHATASE 1A**	ESS073680	2139	-	-	329	F	663	0	Validated
**PROTEIN PHOSPHATASE 2A wbtB**	ESS023456	3762	-	-	634	F	884	0	Validated
**PDH**	ESS011558	422	-	-	79	F	103	2e-27	Validated
**PDH RECEPTOR**	ESS048520	1035	-	-	344	I	359	7e-119	Predicted
**CLOCKWORK ORANGE**	ESS049812	2084	-	-	629	F	226	2e-62	Predicted
**EZH 2**	ESS108699	1719	-	2684	804	F	984	0	Predicted
**HDAC 3**	ESS014944	1677	-	4139	488	F	817	0	Predicted
**CREB binding protein**	ESS048098	2180	-	5322	1301	C	1387	0	Predicted
**TIMELESS 2**	ESS130297	2077	KY923004	4638	1364	F	966	0	Validated

Table S1. Putative *Euphausia superba* circadian protein-encoding transcripts identified via *in silico* transcriptome mining.
Protein type: F = full coding sequence; N = amino-terminal partial protein; I = internal protein fragment; C = carboxy-terminal partial protein.
Sequence status: Confirmed = functionally confirmed component; Validated = sequence validated by molecular cloning; Predicted = *in silico* predicted sequence.

	aa position	Sequence	Description
***Es*CLK**			
NLS	27-58	RNLSEKKRRDQFNLLINELSVMVA ANNRKMDKST	• highly conserved
NES	102-113	LEALDGFFLAI	• highly conserved
***Es*CYC**			
NLS	87-93	EKRRRDK	• highly conserved
NES	112-121	LDKLTVLRM	• highly conserved
***Es*PER**			
NLS	523-532	EPAEKRRRTL	• conserved among crustaceans
	704-739	RHNVDQQHKFVQKQRTHKHSKD RFKIKNI	• conserved in *D. melanogaster,* with only small effect
NES	744-765	KKATKRPHGLKRPGTIMDRECSAP KQIYM	• principal NLS in *D. melanogaster*, not detected by NLSmapper
	502-511	AQNYIVELLA	• principal NES in *D. melanogaster*, the distribution of the hydrophobic residues, albeit not the exact consensus sequence, is conserved in *E. superba*
CLD	426-476	GRPHRSKPYRFRTFNGSYVTLET EWLCFVNPWTMKIDSVIGQHRVIK GPED	• highly conserved
***Es*TIM1**			
NLS	612-621	GPACKRPHHQ	• conserved in *E. superba* and *E. pulchra*
	643-651	RNVKRTKLL	• highly conserved among insects and crustaceans
***Es*CRY2**			
NLS	278-282	RKIKK	• conserved in *M. musculus,* with only small effect

Table S2. Localization signals identified in the main components of krill circadian clock. Abbreviation: NLS = nuclear localization signal; NES = nuclear export signal; CLD = cytoplasmic localization domain.

Figure	Experimental comparison		t-test adjusted p-value
**Fig. 4B**	E-Box-Luc	E-Box-Luc + ***Es*Cyc/Bmal**	0.027
		E-Box-Luc + ***Es*Clk**	0.272
		E-Box-Luc + ***Es*Cyc/Bmal** + **EsClk**	0.004
**Fig. 4B**	E-Box-Luc	E-Box-Luc + ***Es*Cyc/Bmal**	0.008
		E-Box-Luc + ***Es*Clk**	0.016
		E-Box-Luc + ***Es*Cyc/Bmal** + ***Es*Clk**	0.011
**Fig. 4D**	E-Box-Luc + *Es*Cyc/Bmal + *Es*Clk	E-Box-Luc + ***Es*Cyc**$^{1\text{-}629}$+ *Es*Clk	1.7E-04
		E-Box-Luc + *Es*Cyc + ***Es*Clk**$^{1\text{-}1117}$	1.6E-04
		E-Box-Luc + *Es*Cyc + ***Es*Clk**$^{1\text{-}863}$	1.8E-04
		E-Box-Luc + *Es*Cyc + ***Es*Clk**$^{1\text{-}685}$	1.5E-04
		E-Box-Luc + *Es*Cyc + ***Es*Clk**$^{1\text{-}500}$	1.8E-04
	E-Box-Luc + *Es*Cyc + ***Es*Clk**$^{1\text{-}1117}$	E-Box-Luc + *Es*Cyc + ***Es*Clk**$^{1\text{-}863}$	0.025
		E-Box-Luc + *Es*Cyc + ***Es*Clk**$^{1\text{-}685}$	0.051
		E-Box-Luc + *Es*Cyc + ***Es*Clk**$^{1\text{-}500}$	0.935
**Fig. 4E**	E-Box-Luc + *Es*Cyc/Bmal + *Es*Clk	E-Box-Luc + ***d*Cyc +** *Es*Clk	9.9E-05
		E-Box-Luc + *Es*Cyc/Bmal + ***d*Clk**	9.4E-06
	E-Box-Luc + *d*Cyc + *d*Clk	E-Box-Luc + ***d*Cyc +** *Es*Clk	1.2E-05
		E-Box-Luc + *Es*Cyc/Bmal + ***d*Clk**	1.1E-04
**Fig. 4F**	E-Box-Luc + *Es*Cyc/Bmal + *Es*Clk	E-Box-Luc + ***m*Bmal1** + *Es*Clk	9.4E-04
		E-Box-Luc + *Es*Cyc/Bmal + ***m*Clock**	0.007
	E-Box-Luc + *mBmal1* + *m*Clock	E-Box-Luc + ***m*Bmal1** + *Es*Clk	2.0E-04
		E-Box-Luc + *Es*Cyc/Bmal + ***m*Clock**	0.008
**Fig. 5A**	Light<br>E-Box-Luc + *Es*Cyc/Bmal + *Es*Clk	Light<br>E-Box-Luc + *Es*Cyc/Bmal + *Es*Clk + ***Es*Cry1**	0.120
		Light<br>E-Box-Luc + *Es*Cyc/Bmal + *Es*Clk + ***Es*Cry2**	0.002
	Dark<br>E-Box-Luc + *Es*Cyc/Bmal + *Es*Clk	Dark<br>E-Box-Luc + *Es*Cyc/Bmal + *Es*Clk + ***Es*Cry1**	0.050
		Dark<br>E-Box-Luc + *Es*Cyc/Bmal + *Es*Clk + ***Es*Cry2**	9.4E-04
**Fig. 5B**	E-Box-Luc + *Es*Cyc/Bmal + *Es*Clk	E-Box-Luc + *Es*Cyc/Bmal + *Es*Clk + ***Es*Cry1**	1
		E-Box-Luc + *Es*Cyc/Bmal + *Es*Clk + ***Es*Cry2**	0.010
**Fig. 5C**	**Dark**	**Light**	0.007
**Fig. 5D**	E-Box-Luc + *Es*Cyc/Bmal + *Es*Clk	E-Box-Luc + *Es*Cyc/Bmal + *Es*Clk + ***Es*Cry2**	0.002
		E-Box-Luc + *Es*Cyc/Bmal + *Es*Clk + ***Es*Per**	0.062
		E-Box-Luc + *Es*Cyc/Bmal + *Es*Clk + ***Es*Tim1**	0.184
		E-Box-Luc + *Es*Cyc/Bmal + *Es*Clk + ***Es*Per** + ***Es*Tim1**	0.002
		E-Box-Luc + *Es*Cyc/Bmal + *Es*Clk + ***Es*Cry2** + ***Es*Per**	0.002
		E-Box-Luc + *Es*Cyc/Bmal + *Es*Clk + ***Es*Cry2** + ***Es*Tim1**	0.002
	E-Box-Luc + *Es*Cyc/Bmal + *Es*Clk + ***Es*Cry2**	E-Box-Luc + *Es*Cyc/Bmal + *Es*Clk + ***Es*Per** + ***Es*Tim1**	0.035
		E-Box-Luc + *Es*Cyc/Bmal + *Es*Clk + ***Es*Cry2** + ***Es*Per**	0.018
	E-Box-Luc + *Es*Cyc/Bmal + *Es*Clk + ***Es*Per** + ***Es*Tim1**	E-Box-Luc + *Es*Cyc/Bmal + *Es*Clk + ***Es*Cry2** + ***Es*Per**	0.335
	E-Box-Luc + *Es*Cyc/Bmal + *Es*Clk + **1:20 *Es*Cry2**	E-Box-Luc + *Es*Cyc/Bmal + *Es*Clk + 1:20 *Es*Cry2 + ***Es*Per**	3.8E-04
		E-Box-Luc + *Es*Cyc/Bmal + *Es*Clk + 1:20 *Es*Cry2 + ***Es*Tim1**	0.337
		E-Box-Luc + *Es*Cyc/Bmal + *Es*Clk + 1:20 *Es*Cry2 + ***Es*Per + *Es*Tim1**	8.8E-06
	E-Box-Luc + *Es*Cyc/Bmal + *Es*Clk + **1:20 *Es*Cry2 + *Es*Per**	E-Box-Luc + *Es*Cyc/Bmal + *Es*Clk + 1:20 *Es*Cry2 + *Es*Per + ***Es*Tim1**	0.002
	E-Box-Luc + *Es*Cyc/Bmal + *Es*Clk + **1:20 *Es*Per + 1:20 *Es*Tim1**	E-Box-Luc + *Es*Cyc/Bmal + *Es*Clk + 1:20 *Es*Per + ***Es*Tim1**	0.421
		E-Box-Luc + *Es*Cyc/Bmal + *Es*Clk + ***Es*Per +** 1:20 *Es*Tim1	5.2E-05
**Fig. 5E**	E-Box-Luc + *Es*Cyc/Bmal + *Es*Clk	E-Box-Luc + *Es*Cyc/Bmal + *Es*Clk + ***Es*Cry2**	0.003
		E-Box-Luc + *Es*Cyc/Bmal + *Es*Clk + ***Es*Per**	0.442
		E-Box-Luc + *Es*Cyc/Bmal + *Es*Clk + ***Es*Per** + ***Es*Tim1**	0.015
**Fig. 5F**	**IgG control**	**A-V5**	0.002

Table S3. Student's t-test Bonferroni-corrected p-values for all the experimental comparisons discussed.

	CLOCK	CYCLE /BMAL	PERIOD	TIME-LESS	TIME-OUT	Vertebrate-like CRY	*Drosophila* -like CRY	Other CRYs	(6-4)-photolyase	Outgroup
Aedes aegypti	-	AEX32872.1	-	AAY40757.1	XP_001660177.1	-	-	-	-	
Anopheles gambiae	XP_315720.4	XP_556301.3	XP_321212.4	XP_001689006.1	XP_001689129.1	ABB29887.1	XP_321104.4	-	-	
Antheraea pernyi	AAR14936.1	AAR14937.1 (BMAL)	Q17062.1	AAF66996.1	-	ABO38435.1	AAK11644.1	-	-	
Apis mellifera	XP_394233.4	XP_001121441.2 (ARNT-like 1)	NP_001011596.1	-	XP_006565496.1	NP_001077099.1	-	-	-	
Arabidopsis thaliana	-	-	-	-	-	-	-	-	-	NP_567341.1 (Plant CRY)
Bactrocera tryoni	-	-	-	-	-	-	AAU14170.1	-	-	
Blattella germanica	-	-	AAN02439.2	-	-	-	-	-	-	
Bombus impatiens	-	-	-	-	-	NP_001267051.1	-	-	-	
Bombyx mori	-	NP_001036982.1 (CYC-like b)	BAD93177.1	NP_001037622.1	-	NP_001182627.1	NP_001182628.1			
Calanus finmarchicus	comp76772_cl_seq1	compl60482_c0_seq1	compl71214_c0_seq1 (PER1); comp205215_c0_seq1 (PER2)	comp88114_c0_seq1	-	-	comp37700_c0_seq1	-	-	
Ceratitis capitata	-	-	ABB20914.1	-	-	-	-	-	-	
Danaus plexippus	EHJ69324.1	AAR13012.1	EHJ74075.1	EHJ67997.1	EHJ76705.1	ABA62409.1	AAX58599.1	-	-	
Danio rerio	AAI63244.1	NP_571652.1 (ARNT-like 1)	AAO38747.1 (PER2)	-	NP_001265529.1	NP_001070765.2 (CRY1a); NP_571865.4 (CRY1b); BAA96848.1 (CRY2a); BAA96849.1 (CRY2b); BAA96850.1 (CRY3)	-	AAH44385.1 (CRY4)	AAH44204.1 (CRY5)	
Daphnia magna	-	-	-	JAN75617.1	JAN88373.1	-	KZS20490.1	-	-	
Daphnia pulex	NCBI_GNO_118274	NCBI_GNO_1144014	NCBI_GNO_212404	NCBI_GNO_238244	NCBI_GNO_88254	EFX82092.1	EFX77441.1	-	-	
Dianemobius nigrofasciatus	-	-	-	-	-	-	BAF45421.1	-	-	
Drosophila melanogaster	NP_523964.2 (CLK isoform A)	NP_524168.2	NP_525056.2	AAC46920.1	AAF73481.1	-	NP_732407.1	-	NP_477188.1	NP_731308.1 (TGO isoform A)
Eurydice pulchra	AGV28720.1 (CLK5)	AGV28715.1 (ARNT-like 1)	AGV28714.1	AGV28716.1	-	AGV28717.1	-	-	-	
Gallus gallus	AAL98708.1	NP_001001463.1	NP_989593.1 (PER2)	-	-	NP_989576.1 (CRY1); NP_989575.1 (CRY2)	-	NP_001034685.1 (CRY4)	-	
Gryllus bimaculatus	BAM76759.1	BAN28450.1	BAG48878.1	BAJ16356.1	-	-	-	-	-	
Homo sapiens	AAF13733.1	BAA19935.1 (BMAL1b)	NP_073728.1 (PER2)	-	BAA36499.1	NP_004066.1 (CRY1); NP_001120929.1 (CRY2)	-	-	-	NP_001612.1 (ARNT)
Macrobrachium rosenbergii	AAX44045.1	-	-	-	-	-	-	-	-	
Mamestra brassicae	-	-	-	-	-	-	AAY23345.1	-	-	
Mus musculus	NP_031741.1 (CLOCK isoform 1)	BAD26600.1 (BMAL1b)	NP_035196.2 (PER2)	-	NP_001129554.1	NP_031797.1 (CRY1); NP_034093.1 (CRY2)	-	-	-	
Nephrops norvegicus	-	-	ALC74274.1	-	-	-	-	-	-	
Neurospora crassa	-	-	-	-	-	-	-	-	-	XP_958771.1 (TOF1)
Pacifastacus leniusculus	-	AFV39705.1 (BMAL1a)	-	-	-	-	-	-	-	
Talitrus saltator	-	-	-	-	-	AFV96168.1	-	-	-	
Thermobia domestica	-	BAJ16354.1	-	-	-	-	-	-	-	
Tigriopus californicus	-	JW523145	JW535312	JW538330	-	-	-	-	-	
Tribolium castaneum	XP_008199501.1 (CLK isoform X1)	NP_001107795.1	NP_001106933.1	NP_001106934.1	XP_008201051.1	NP_001076794.1	-	-	-	
Xenopus laevis	NP_001083854.2	AAW80970.1 (BMAL1)	NP_001081098.1 (PER2)	-	XP_018102910.1	NP_001081129.1 (CRY1); NP_001082139.1 (CRY2a); AAK94667.1 (CRY2b)	-	-	-	

Table S4. Accession numbers for protein sequences included in the phylogenetic analyses. Protein sequences of the molecular clock components from *Drosophila melanogaster*, *Apis mellifera*, *Antheraea pernyi*, *Danaus plexippus*, *Daphnia pulex*, *Macrobrachium rosenbergii*, *Euridice pulchra*, and *Tigriopus californicus* were used to query the database. Orthologues to *E. superba* clock components were retrieved from NCBI databases (https://www.ncbi.nlm.nih.gov). For *Daphnia pulex*, protein sequences were downloaded from FleaBase (http://wfleabase.org). For *Calanus finmarchicus* we used the contigs published by Christie *et al.* (9)**.** Legend**: -** = not used or not available.

Construct Name	Vector	Enzymatic digestion	Tag	Tag position	Expressed proteins	Primers (5'->3')
p*Es*Clk	pAc5.1/V5-HisA	EcoRI; NotI	FLAG	C-ter	*Es*CLOCK/FLAG	**F:**CAGTGTGGTGGAATTCATGTACGAAGAAGAAAGCATT<br>**R:**TAGACTCGAGCGGCCGCTTACTTGTCGTCATCGTCTTTGTAGTCCTTCTTGGGTGGTAGCTGTGG
p*Es*Cyc/Bmal	pAc5-STABLE2-neo	EcoRI; NotI	His	C-ter	*Es*CYCLE/His; GFP; neo	**F:**CAGTGTGGTGGAATTCATGTTCGGTCTGGGGAATTAT<br>**R:**GCGTCCTTCGCGGCCGCCGTGGTGGTGGTGGTGGTGTGGCAGTGGCCATGGCAGATG
p*d*Clk	pAc5.1/V5-HisA	EcoRI; NotI	FLAG	C-ter	*d*CLOCK/FLAG	**F:**CAGTGTGGTGGAATTCATGGACGACGAGAGCGACGAC<br>**R:**TAGACTCGAGCGGCCGCTTACTTGTCGTCATCGTCTTTGTAGTCTTGACTACTGCCTGGGGCTGT
p*d*Cyc	pAc5-STABLE2-neo	EcoRI; NotI	His	C-ter	*d*CYCLE/His; GFP; neo	**F:**CAGTGTGGTGGAATTCATGGAAGTTCAGGAGTTCTGC<br>**R:**GCGTCCTTCGCGGCCGCCGTGGTGGTGGTGGTGGTGTAAGAAGACGGAGTTCTTGGC
p*Es*Clk$^{1-1117}$	pAc5.1/V5-HisA	EcoRI; NotI	FLAG	C-ter	*Es*CLOCK$^{1-1117}$/ FLAG	**F:**CAGTGTGGTGGAATTCATGTACGAAGAAGAAAGCATT<br>**R:**TAGACTCGAGCGGCCGCTTACTTGTCGTCATCGTCTTTGTAGTCTTGGGGTAGCTGCACGGACAA
p*Es*Clk$^{1-863}$	pAc5.1/V5-HisA	EcoRI; NotI	FLAG	C-ter	*Es*CLOCK$^{1-863}$/ FLAG	**F:**CAGTGTGGTGGAATTCATGTACGAAGAAGAAAGCATT<br>**R:**TAGACTCGAGCGGCCGCTTACTTGTCGTCATCGTCTTTGTAGTCGCTGACCATTGATCCCATAAG
p*Es*Clk$^{1-685}$	pAc5.1/V5-HisA	EcoRI; NotI	FLAG	C-ter	*Es*CLOCK$^{1-685}$/ FLAG	**F:**CAGTGTGGTGGAATTCATGTACGAAGAAGAAAGCATT<br>**R:**TAGACTCGAGCGGCCGCTTACTTGTCGTCATCGTCTTTGTAGTCGTCTCCTCTTGATGGCAGGTG
p*Es*Clk$^{1-500}$	pAc5.1/V5-HisA	EcoRI; NotI	FLAG	C-ter	*Es*CLOCK$^{1-500}$/ FLAG	**F:**CAGTGTGGTGGAATTCATGTACGAAGAAGAAAGCATT<br>**R:**TAGACTCGAGCGGCCGCTTACTTGTCGTCATCGTCTTTGTAGTCGTCCGCTATGGATCTACGTTG
p*Es*Cyc/ Bmal$^{1-629}$	pAc5-STABLE2-neo	EcoRI; NotI	His	C-ter	*Es*Cyc/Bmal$^{1-629}$/ His; GFP; neo	**F:**CAGTGTGGTGGAATTCATGTTCGGTCTGGGGAATTAT<br>**R:**GCGTCCTTCGCGGCCGCCGTGGTGGTGGTGGTGGTGCGCCCCGCCCCCATCACCTGC
p*Es*Per	pAc5-STABLE2-neo	EcoRI; NotI	Myc	C-ter	*Es*PERIOD/Myc; GFP; neo	**F:**CAGTGTGGTGGAATTCATGACTGAAAATGAGAGATCT<br>**R:**GCGTCCTTCGCGGCCGCCCAGATCCTCTTCTGAGATGAGTTTTTGTTCTGAGTCGGAGGAGTCTCTACC
p*Es*Tim	pAc5.1/V5-HisA	EcoRI; NotI	V5	C-ter	*Es*TIMELESS/ V5	**F:**CAGTGTGGTGGAATTCATGGAGTGGATGATGATGAAC<br>**R:**TAGACTCGAGCGGCCGCTTACGTAGAATCGAGACCGAGGAGAGGGTTAGGGATAGGCTTACCGTGTAAGTGAGCTGGCGCAGG
p*Es*Cry1	pAc5.1/V5-HisA	EcoRI; NotI	HA	C-ter	*Es*CRY1/HA	**F:**TCCAGTGTGGTGGAATTCATGACCAACACTGGTTGTGTAG<br>**R:**CGGCCGCCACTCGAGTTAAGCGTAATCTGGAACATCGTATGGGTAACAGACATTGTGTTGACATTGATC
p*Es*Cry2	pAc5.1/V5-HisA	EcoRI; NotI	Myc	C-ter	*Es*CRY2/Myc	**F:**TCCAGTGTGGTGGAATTCATGACGAGAGGAGGAGAGAAACATG<br>**R:**CGGCCGCCACTCGAGTTAAGCGTAATCTGGAACATCGTATGGGTAGGCCGGCTGCATTTGCTGTGT

Table S5. Main features for the generation of the constructs used for the functional characterization of the krill circadian clock components.

Transcript	Forward Primer (5'->3')	Reverse Primer (5'->3')
*Es*Clock	GCTGCAGCAAATGATAATGC	TGCCATATTGGGCCATAACT
*Es*Cycle	TTTTGATTTAAGGCATTTTTCATCTA	CCCAGTCTCACGATTCTAGCTACT
*Es*Period	GCAGCTATGCCCAACTTTAATC	GGACTAGCAACAGGGACATTTT
*Es*Timeless1	TTGAAGACTCTCCTACAATGATGG	TACAGAGGCCGCATGACA
*Es*Cryptochrome2	TCATGAACCATGGACTGCAC	GGTGGACACGACTTCAACAA
Spike	AATCCCCGACTCAGACCCAT	GGCTTCGGGAAATCAGCTGA

Spike sequence	TGATGGATGCCACCAATATGCCAGCTGTCAAAGCCATCATATATCAGTATATGGAAGAGATTTATCATAGAA TCCCCGACTCAGACCCATCTTCTAGCAAAACAAGGCAGATCATCTCCACCATCCGGACGCAGAATCTTCCC AACTGTCAGCTGATTTCCCGAAGCCACTACTCCCCCATCTACCTGTCGTTTGTCA

Table S6. Primers for quantitative real-time PCR experiments and spike synthetic oligoribonucleotide sequence.
